# *In silico* Identification and Expression of Protocadherin Gene Family in *Octopus vulgaris*

**DOI:** 10.3389/fphys.2018.01905

**Published:** 2019-01-14

**Authors:** Ruth Styfhals, Eve Seuntjens, Oleg Simakov, Remo Sanges, Graziano Fiorito

**Affiliations:** ^1^Department of Biology and Evolution of Marine Organisms, Stazione Zoologica Anton Dohrn, Naples, Italy; ^2^Laboratory of Developmental Neurobiology, Department of Biology, KU Leuven, Leuven, Belgium; ^3^Department of Molecular Evolution and Development, University of Vienna, Vienna, Austria; ^4^Computational Genomics Laboratory, Neuroscience Area, International School for Advanced Studies (SISSA), Trieste, Italy

**Keywords:** protocadherins, DSCAM, plasticity, neural wiring, octopus, cephalopod

## Abstract

Connecting millions of neurons to create a functional neural circuit is a daunting challenge. Vertebrates developed a molecular system at the cell membrane to allow neurons to recognize each other by distinguishing self from non-self through homophilic protocadherin interactions. In mammals, the protocadherin gene family counts about 50 different genes. By hetero-multimerization, protocadherins are capable of generating an impressive number of molecular interfaces. Surprisingly, in the California two-spot octopus, *Octopus bimaculoides*, an invertebrate belonging to the Phylum Mollusca, over 160 protocadherins (PCDHs) have been identified. Here we briefly discuss the role of PCDHs in neural wiring and conduct a comparative study of the protocadherin gene family in two closely related octopus species, *Octopus vulgaris* and *O. bimaculoides*. A first glance at the expression patterns of protocadherins in *O. vulgaris* is also provided. Finally, we comment on PCDH evolution in the light of invertebrate nervous system plasticity.

## Neural Wiring and Neuronal Recognition: Protocadherins and Down Syndrome Cell Adhesion Molecule

Neurons are capable of recognizing each other through a neuronal barcode-like mechanism (i.e., chemoaffinity hypothesis, Sperry, 1963). The establishment of a molecular identity allows neurons to form connections with appropriate “partners” and to discriminate self from non-self, an essential feature to build-up neural networks during development and/or structural remodeling in the adult (Christensen et al., 2013; Schreiner et al., 2017). Various molecules such as the immunoglobulins and cadherins have been implicated in this synaptic specificity (de Wit and Ghosh, 2016).

### The Protocadherin Gene Family

Protocadherins (PCDHs) are cell-adhesion molecules and represent the largest subgroup of the cadherin superfamily. PCDHs contain six or seven extracellular cadherin (EC) repeats, and are considered a chordate innovation (Hulpiau and van Roy, 2011). They are expressed mainly in the nervous system and seem to be involved in both nervous system development and functioning (reviewed by Peek et al., 2017). The majority of mammalian PCDHs are located together on the genome in three gene clusters (i.e., PCDHa, PCDHb, PCDHg; for review see Hirayama and Yagi, 2017). It has been suggested that vertebrates utilize clustered PCDHs to generate neuronal identities essential for synaptic specificity. For instance, the differential expression of PCDHs, through alternative promoter choice and tetramerization at the cell surface, allows the 22 PCDHγ genes to generate over 234,256 different extracellular regions (Schreiner and Weiner, 2010). The non-clustered protocadherins are scattered throughout the genome. They are expressed in specific neural regions in the mammalian brain, while the clustered PCDHs are broadly expressed throughout various brain regions, although they exhibit a certain cell-type specificity (e.g., Zou et al., 2007). The observed PCDH expression patterns are related to their function; the non-clustered PCDHs are known to be involved during early stages such as axon outgrowth and path-finding, while clustered PCDHs are essential for axon terminal formation and dendritic self-avoidance, thus helping the establishment of neural-specific connections (Goodman et al., 2017; Peek et al., 2017).

PCDHs are also known to be continuously expressed in adult mammalian brains, with elevated expression levels in the hippocampus, cerebellum and cortex (e.g., Hertel et al., 2008, 2012; Junghans et al., 2008; Nuernberger et al., 2008; Kim et al., 2010; Krishna-K et al., 2011), suggesting a role in adult brain functioning, beyond the establishment of neural connectivity.

### DSCAM, an Alternative to PCDHs in Invertebrates

In the insect *Drosophila melanogaster* protocadherins found their counterpart in the repertoire of DSCAM (Down syndrome cell adhesion molecule) isoforms. While *D. melanogaster* lacks PCDHs completely, over 19,008 unique DSCAM isoforms are formed through extensive alternative splicing (Schmucker et al., 2000; Schmucker and Chen, 2009; Zipursky and Sanes, 2010). *D. melanogaster* DSCAMs act in the recognition of neural self vs. non-self (Hattori et al., 2008). DSCAM is known to be required for axon guidance and for the formation of axon pathways in the nervous system, and their molecular diversity is suggested to contribute to the specificity of neuronal connectivity (Schmucker et al., 2000; Hummel et al., 2003; Zhan et al., 2004; Zhu et al., 2006; Matthews et al., 2007). In analogy to what is known for clustered PCDHs, axons expressing the same set of DSCAM isoforms repel each other, thus ensuring neural branch segregation (Zhan et al., 2004). Intriguingly, the arthropods developed two different molecular mechanisms to generate neuronal diversity. Hexapods and crustaceans possess the same hypervariable DSCAM gene, and isoforms are generated as in *D. melanogaster* (Brites et al., 2008; Armitage et al., 2012). In contrast, in Chelicerata DSCAM developed a genomic organization similar to vertebrate PCDHs, which arose through duplication events (Yue et al., 2016). Instead of generating Chelicerata-DSCAM isoforms through splicing, different DSCAMs are expressed through alternative promoter choice (Cao et al., 2018).

Which mechanism a species uses to generate their repertoire of cell-recognition molecules, therefore, appears less important. What seems to be more essential is the available number of cell-recognition molecules and how these molecules convey the signal that is generated upon cell-cell interaction. The similarities on a functional, genomic and molecular level between the clustered protocadherins and the Chelicerata-DSCAM are highly intriguing considering the fact that these proteins share no sequence homology (for review see Jin and Li, 2018).

## Protocadherins: a Short Overview Throughout the Animal Kingdom

The protocadherin gene clusters are considered to be a vertebrate innovation and their diversity among species (i.e., lineage-specific duplication, gene conversion, adaptive variation in diversified ectodomains) has been suggested to drive the substantial increase in central nervous system complexity in vertebrates relative to other species (Noonan et al., 2004b).

The human genome contains 12 non-clustered and 53 clustered PCDHs. Although mammalian protocadherins are known to be orthologous, differences can even be found between humans and chimpanzees. Open reading frame-changing nucleotide insertions in no less than three PCDH genes have been found (Wu, 2005). Sequence differences among orthologous PCDHs in several vertebrate lineages appear to reflect adaptive differences in protocadherin function that contribute to clade-specific structural and functional specializations of the nervous system.

Protocadherins in humans, mice, rats, lizards, elephant sharks, and coelacanths are similarly organized in 3–4 clusters on a single locus (Wu and Maniatis, 1999; Noonan et al., 2004a; Yu et al., 2008; Jiang et al., 2009; but see for *Xenopus* Etlioglu et al., 2016). Teleosts exhibit an intriguing increase in clustered PCDHs due to fish-specific whole genome duplications (Wu, 2005; Yu et al., 2008). Their genomes contain two PCDHα and two PCDHγ clusters located on two different loci, but lack the PCDHβ cluster completely. Until recently, it was thought that clustered genomic organization was maintained throughout vertebrate evolution. However, cyclostomes are known to possess only non-clustered protocadherins (Ravi et al., 2015).

**FIGURE 1:**
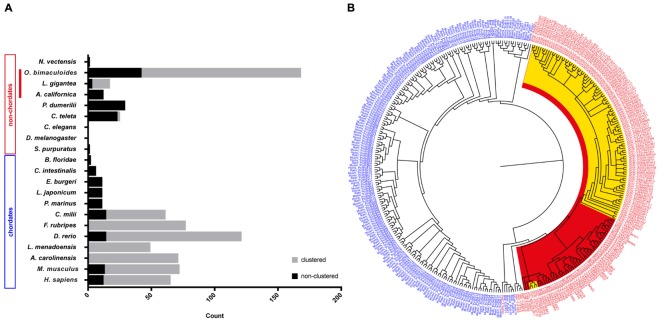
Distribution and evolution of the protocadherin gene family in metazoans. **(A)** Abundance of protocadherins in the genomes of different chordate and non-chordate species. Data are derived from: Wu and Maniatis (1999), Noonan et al. (2004a,b), Wu (2005), Whittaker et al. (2006), Yu et al. (2007), Noda and Satoh (2008), Yu et al. (2008), Jiang et al. (2009), Hulpiau and van Roy (2011), Albertin et al. (2015), and Ravi et al. (2015). The attribution to clustered vs. non-clustered PCDHs in the graph (*Lottia gigantea* and *Octopus bimaculoides*) is derived from Authors’ estimation (Albertin et al., 2015). **(B)** Bayesian phylogenetic reconstruction of the evolutionary relationships between protocadherins in different species. Chordate PCDHs are visualized in blue and non-chordate PCDHs are shown in red. Molluscan PCDHs are highlighted in red. The included cephalopod species are *Octopus vulgaris* and *O. bimaculoides*, which are highlighted in yellow. Octopus protocadherins interdigitate on the tree (see Supplementary Figure S2).

In an attempt to summarize the relative distribution of PCDHs in the animal kingdom, we surveyed data in the literature to illustrate the relative abundance of protocadherins in different chordate and non-chordate species (Figure 1A). In invertebrates, only low abundances (or absence) of protocadherins have been detected in the genomes of several species such as *Lottia gigantea*, *Caenorhabditis elegans*, *D. melanogaster*, *Ciona intestinalis*, and *Strongylocentrotus purpuratus* to mention some (Figure 1A). The PCDHs identified in the genomes of invertebrates are generally non-clustered protocadherins, the exceptions being *L. gigantea* and cephalopods (see below).

## Cephalopod Protocadherins

The recent genome sequencing of the cephalopod mollusc *Octopus bimaculoides* (Albertin et al., 2015) and the data provided for the Longfin inshore squid *Doryteuthis pealeii* identified a large amount of clustered PCDH in cephalopods (Albertin et al., 2015; see also Wang and Ragsdale, 2017). In particular, the *O. bimaculoides* genome was found to encode over 120 clustered protocadherins and about 50 non-clustered PCDHs (168 multi-exonic PCDH genes, Albertin et al., 2015). Furthermore, 155 PCDHs have been identified in transcriptomes of the squid *D. pealeii* (Albertin et al., 2015). Interestingly, they showed that the expansion of protocadherins occurred independently in squid and octopus (Albertin et al., 2015). Octopus PCDHs are characterized as clustering together on the genome, an organization that includes a head-to-tail arrangement, analogous to what has been documented in the case of mammalian clustered PCDHs (Chen and Maniatis, 2013; Wang and Ragsdale, 2017). According to the summary provided by Wang and Ragsdale (2017), the three largest octopus clusters comprise 31, 17, and 10 PCDHs, while more than twenty scaffolds include at least two protocadherins. In their analysis of the known intracellular domain-motifs in octopus PCDHs, they were unable to find any analogy with vertebrates. Expression analysis showed that Ob-PCDHs are particularly enriched within the nervous system, mainly within the optic lobes and the axial nerve cord (Albertin et al., 2015; Wang and Ragsdale, 2017). It is also intriguing that cadherins have been identified in *O. bimaculoides* including one with 77 EC domains, that appears highly expressed in octopus suckers (Wang and Ragsdale, 2017).

Recent *de novo* transcriptomes of other cephalopod species (i.e., *S. officinalis, Octopus vulgaris* and *O. bimaculoides*) have provided evidence of a variable but large number (spanning from 127 to 251) of protocadherin open reading frames (Liscovitch-Brauer et al., 2017). This work confirms also previous recent evidence of the existence of RNA-editing in cephalopods, and suggests that RNA-editing is more extensive in protocadherins with respect to other genes in cephalopods. Interestingly, in the transcriptome of *Nautilus*, which had significantly less RNA editing sites, only 28 PCDH open reading frames have been recognized (Liscovitch-Brauer et al., 2017). We speculate that the very low abundance of PCDHs within *Nautilus* may simply reflect a less complex nervous system within the cephalopod clade (Nixon and Young, 2003). In *Callistoctopus minor* over 300 genes are reported as protocadherins (Kim et al., 2018).

The above-mentioned account of the PCDH gene family expansion in one representative taxon of the Lophotrochozoa, i.e., cephalopods (Albertin et al., 2015), *de facto* challenges the view that protocadherins are a vertebrate innovation (Yu et al., 2008). It seems that protocadherins expanded independently in two very distant clades, namely Lophotrochozoa and Vertebrata. This is confirmed by the enrichment of protocadherins in the nervous system of both coleoid cephalopods and vertebrates, representing a striking case of convergent evolution.

### PCDHs in the Common Octopus, *Octopus vulgaris*

To further contribute to the knowledge of PCDHs in cephalopods, we explored the available transcriptome of another cephalopod species, the common octopus *O. vulgaris*, obtained from the research groups of Drs. R. Sanges and G. Fiorito at the Stazione Zoologica Anton Dohrn, Naples, Italy. The *O. vulgaris* transcriptome was based on RNA-Seq studies carried out on the central nervous system (i.e., optic lobes, supra-esophageal and sub-esophageal masses), proximal and distal extremities of arm (including muscular and/or nervous tissues), and other nervous system ganglia (Petrosino, 2015). The resulting transcriptome identified more than a hundred thousand expressed transcripts from different neural structures, significantly extending previously available transcriptome data for this species (Zhang et al., 2012; but see also Liscovitch-Brauer et al., 2017). By mining the *O. vulgaris* transcriptome for sequences containing four, five, six, or seven cadherin repeats, we identified 53 unique putative protocadherin gene sequences which can be used for future gene expression analysis (see Supplementary Information). This number is likely an underestimation, given the stringency of the analysis and the fact that we relied on a transcriptome assembly.

A phylogenetic tree of PCDHs comparing different vertebrate and invertebrate species, illustrates that the PCDH repertoire in two different octopus species (*O. vulgaris* and *O. bimaculoides*; characterized by different life cycles) did not evolve independently. The PCDH expansion occurred before speciation in octopus, thus suggesting that they are orthologous (Figure 1B and Supplementary Figure S2). The clustered Ob-PCDHs have extremely similar sequences, which is possibly due to recent gene duplications or gene conversions (Albertin et al., 2015). In addition, the specific phylogenetic tree of *O. vulgaris* (Supplementary Figure S3) shows that protocadherins possessing seven EC repeats are significantly different from Ov-PCDH possessing less repeats, which is reminiscent of the non-clustered δ1-PCDH subfamily in vertebrates. It would be interesting to see whether this convergence also exists at a genomic and functional level.

Moreover, two of these δ1-PCDH-like genes seem to cluster together with other molluscs (Figure 1B and Supplementary Figure S2), which would suggest that they are ancestral to other Ov-PCDHs. This observation supports the previous hypothesis that ancient PCDHs possessed more EC domains, which got lost or rearranged during evolution (Hulpiau and van Roy, 2011). Various Ov-PCDH and Ob-PCDH seem to possess very short extracellular regions (5 EC, data not shown) compared to vertebrate PCDHs (6 EC or 7 EC). According to Hulpiau and van Roy (2011), this would suggest that short octopus PCDHs are more evolved than those of the vertebrates.

Not much is known about the intracellular partners of PCDHs in vertebrates (Weiner and Jontes, 2013). Whether these intracellular interactions are conserved in *O. vulgaris* remains unexplored. Based on our current data, we have no evidence for the presence of the cytoplasmic domains that characterize vertebrate δ1-PCDH (CM1, CM2, CM3) in *O. vulgaris*, thus suggesting that Ov-PCDHs may have developed different intracellular pathways (see Supplementary Information). Octopus-specific motifs identified by Albertin et al. (2015) were found in the Ov-PCDHs (Supplementary Table S1).

Based upon the presence of cadherin repeats, we propose conserved extracellular interactions of Ov-PCDH. It is probable that they will act as cell-adhesion molecules, although nothing is known regarding their adhesion specificity. After alignment of the first EC repeat, we found around 30% identity with vertebrate protocadherins at the protein level, an expected value for non-orthologous proteins (see Supplementary Table S1 and Supplementary Figure S5). Based upon alignment of Ov-PCDH transcripts we show around 98% identity at a protein level between protocadherins in *O. vulgaris* and *O. bimaculoides* (See Supplementary Information: Sequence Alignments).

**FIGURE 2:**
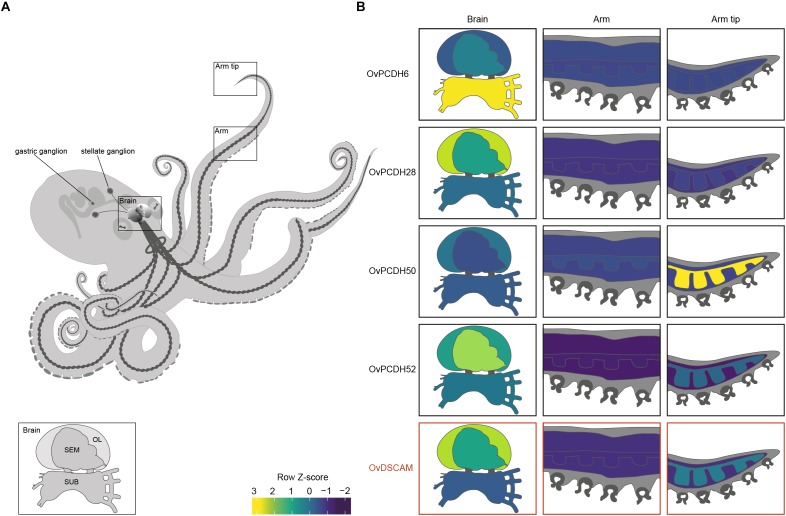
Protocadherin and Dscam expression in *O. vulgaris.* **(A)** A schematic overview of the octopus and the main components of its nervous system. The octopus brain (SEM: supra-esophageal mass; SUB: sub-esophageal mass; OL: optic lobe), arm nerve cord, stellate and gastric ganglia are visualized. **(B)** Relative expression levels (coded according to Row Z-score) of selected Ov-PCDHs (see Supplementary Information and Supplementary Figure S1) and Ov-DSCAM are shown in the brain (supra-esophageal mass; sub-esophageal mass; optic lobe), arm (muscle tissue and axial nerve cord) and arm tip.

Previous PCDH expression analysis in Albertin et al. (2015) showed increased expression within the nervous system, suggesting that cephalopod protocadherins play an important role in the nervous system of these organisms. The same disparity can be observed between neural and non-neural tissue in *O. vulgaris* (Figure 2 and Supplementary Figure S1). As in vertebrates, we found few PCDHs expressed in non-neural octopus tissues. Our findings, based on *in silico* data, highlight the lower expression in the sub-esophageal mass, possibly explained by the presence of fewer neurons in comparison to the supra-esophageal mass and the optic lobes. However, it is also possible that less active reorganization of the neural circuitry is required in adults within brain areas controlling basic motor patterns. We also found an elevated PCDH expression of three different genes (6 or 7 EC) in the arm tip (Figure 2 and Supplementary Figure S1), a region that may require continuous growth and rewiring of newly developing sensory systems. Moreover, protocadherins appear differentially expressed in the supra-esophageal mass, sub-esophageal mass, optic lobe and the stellate ganglion of *O. vulgaris*.

### Octopus DSCAM

We applied the same strategy (see Supplementary Information) for the identification of DSCAM in *O. vulgaris*. Our assembled *O. vulgaris* transcriptome possesses only one DSCAM transcript, while the genome of *O. bimaculoides* presents two different isoforms of the same gene.

Based on the phylogenetic reconstruction, octopus DSCAM shows close identity with DSCAMs in other molluscs (Supplementary Figure S4). Ov-DSCAM and Ov-PCDHs have similar expression patterns throughout the nervous system of *O. vulgaris* (Figure 2 and Supplementary Figure S1). It is speculated here that Ov-DSCAM has a similar role to vertebrate DSCAM, and exerts its function in a complementary manner to the PCDH gene clusters. DSCAM and DSCAM-L1 in vertebrates have been shown to be essential for neurite self-avoidance, but not for synaptic specificity (Fuerst et al., 2009).

## Closing Remarks

Here we show for the first time the presence of a large number of protocadherins in the transcriptome of the cephalopod mollusc *O. vulgaris*. Our data reveal the existence of differential expression of PCDHs in different brain lobes of the nervous system of an adult octopus. The increased expression of some PCDHs in the supra-esophageal mass and the optic lobes is intriguing since these are the areas where most of neural computation is achieved, including processes such as learning and memory (Young, 1991; Hochner et al., 2006; Marini et al., 2017; Turchetti-Maia et al., 2017).

Based on the expression of protocadherins in brain of adult mammals, such as the hippocampus and cerebellum (e.g., Hertel et al., 2008, 2012; Junghans et al., 2008; Nuernberger et al., 2008; Kim et al., 2010; Krishna-K et al., 2011), we propose a role for protocadherins in adult octopus brain functioning. Several examples are available in support of the hypothesis that PCDHs are involved in neural plasticity. First, electroconvulsive shocks induce neural activity evoking structural rearrangements through neurogenesis and synaptogenesis (Scott et al., 2000), as well as altered non-clustered PCDH-gene expression (Kim et al., 2010). Second, several non-clustered PCDHs, belonging to the δ1-subgroup, are known to affect synaptic plasticity through a conserved motif “RRVTF” in their cytoplasmic domain (Vanhalst et al., 2005). Protein phosphatase1-α specifically binds to this motif, thereby regulating synaptic plasticity at three different levels (for review see: Winder and Sweatt, 2001; Vanhalst et al., 2005). Third, an antibody against Arcadlin, the rat homolog of PCDH8, interfered with long-term potentiation in slice preparations of the rat hippocampus (Yamagata et al., 1999). Fourth, PCDH10 has been implicated in complex molecular cascades regulating synapse elimination in the mouse hippocampus (Tsai et al., 2012). Additionally, the intracellular domain of PCDHα genes can interact with a tyrosine kinase, *fyn* (Kohmura et al., 1998). In the mouse hippocampus, *fyn* is involved in inducing NMDA receptor-dependent long-term potentiation (Grant et al., 1992). Last but not least, the human-specific gene pair PCDH11X/Y has been recognized to play a role in the development of human language (Speevak and Farrell, 2011; Priddle and Crow, 2012, 2013). To the best of our knowledge, the examples provided above represent known cases of vertebrate PCDH involvement in neural plasticity. Furthermore, synaptic activity has been shown to modulate protein turnover, which allows change and thus plasticity at the level of the synapse (Bingol and Sheng, 2011; Alvarez-Castelao and Schuman, 2015; Cohen and Ziv, 2017). We suggest that synaptic plasticity can be achieved through PCDH synthesis and degradation. By replacing the protocadherin repertoire at its cell surface, each neuron would be theoretically capable of forming new synaptic connections, thereby mediating structural plasticity in the adult (de Wit and Ghosh, 2016).

We speculate that the expansion of the protocadherin gene family in vertebrates and in cephalopods can be linked to the development of brain complexity and the increased plasticity in the adult brains. Uncovering expression patterns of both DSCAM and PCDHs in octopus will yield insights into their potential function. We expect that protocadherins that are involved in synaptic specificity will be expressed in a mosaic pattern distributed across the entire brain, whereas localized expression suggests a role in target recognition and axonal outgrowth. This seems to be the case in *O. vulgaris* since differential expression can be observed in different brain regions (Figure 2 and Supplementary Figure S1). In addition to elucidation and analysis of these patterns in various parts of the octopus brain (e.g., the supra-esophageal mass and the optic lobes), the investigation of PCDH expression patterns during development and regeneration in *O. vulgaris* (e.g., Imperadore et al., 2017; Zullo et al., 2017) will be central in future studies.

The increased expression of a number of protocadherins in the stellate ganglion of *O. vulgaris* suggests that PCDHs are involved in plasticity related to the neural control of the chromatophores; key neuro-muscular organs involved in body patterning.

The biological role of DSCAM in the octopus is also an interesting problem whose future elucidation may facilitate comparative evolutionary analysis.

Finally, the putative differential expression of different PCDHs in octopus (and cephalopods generally) opens up a new avenue of studies aimed at deciphering the contribution of these adhesion molecules to neural wiring and neural plasticity in the adult, as in the case of the higher vertebrates.

## Ethics Statement

The study is *in silico* only, based on samples obtained from *Octopus vulgaris* RNA-seq experiments collected in 2011 and 2012, thus well before the entry into force of Directive 2010/63/EU.

## Author Contributions

RSt, ES, and GF conceived this manuscript. RSt carried out the analysis and drafted the manuscript. OS and RSa provided guidance on the bioinformatic analysis. All authors discussed the results, contributed to writing and commented on the manuscript at all stages, and read and approved the submitted manuscript.

## Conflict of Interest Statement

The authors declare that the research was conducted in the absence of any commercial or financial relationships that could be construed as a potential conflict of interest.
