# Supplementary material for: In silico Identification and Expression of Protocadherin Gene Family in Octopus vulgaris
Source: Front Physiol. 2019 Jan 14;9:1905. doi: 10.3389/fphys.2018.01905 (PMC6339937; doi:10.3389/fphys.2018.01905)
Supplement: Supplementary file 1 [file Data_Sheet_1.pdf]

In silico identification and expression of protocadherin gene family in *Octopus vulgaris*

Supplementary Information

Ruth Styfhals[1,2], Eve Seuntjens[2], Oleg Simakov[3], Remo Sanges[1,4], Graziano Fiorito[1]

[1] Department of Biology and Evolution of Marine Organisms, Stazione Zoologica Anton Dohrn Napoli, Italy

[2] Laboratory of Developmental Neurobiology, Department of Biology, KU Leuven, Belgium

[3] Department of Molecular Evolution and Development, University of Vienna, Austria

[4] Computational Genomics Laboratory, Neuroscience Area, International School for Advanced Studies (SISSA), Trieste, Italy

Corresponding Author:
Ruth Styfhals
Department of Biology and Evolution of Marine Organisms, Stazione Zoologica Anton Dohrn Napoli, Italy
Email: ruth.styfhals@szn.it

Keywords: Protocadherins, DSCAM, plasticity, neural wiring, octopus, cephalopods

Table of Contents

METHODS **3**
ANNOTATION 3
EXPRESSION ANALYSIS 4
PHYLOGENETIC RECONSTRUCTION 5
SEQUENCE ANALYSIS 5
RESULTS **6**
SEQUENCE ALIGNMENTS **13**
Ov-PCDH6-c36174_g3_i1 13
Ov-PCDH28-c35066_g15_i1 16
Ov-PCDH50-c32730_g4_i1 19
Ov-PCDH52-c31207_g1_i5 21
Ov-DSCAM-c34599_g6_i1 24
ACCESSION NUMBERS **32**
REFERENCES **34**

Methods

Annotation

We identified putative PCDHs and DSCAM sequences in the transcriptome of *O. vulgaris* (G. Petrosino, G. Ponte, R. Sanges and G. Fiorito, pers. communication). The *O. vulgaris* transcriptome has been based on RNA-seq studies (Petrosino, 2015) carried out on *O. vulgaris* central nervous system (i.e., optic lobes, supra-esophageal and sub-esophageal masses), proximal and distal extremities of arm (including muscular and/or nervous tissues), and other nervous system ganglia. The resulting transcriptome identified about a hundred thousand transcripts from different neural structures, significantly extending previously available transcriptome data for the brain of this species (Zhang et al., 2012; but see Liscovitch-Brauer et al., 2017).

We used known protocadherin protein sequences of several species: *Homo sapiens, Mus musculus, Danio rerio, Branchiostoma floridae, Ciona intestinalis, Strongylocentrotus purpuratus, Tribolium castaneum, Capitella teleta, Platynereis dumerilii, Aplysia californica Lottia gigantea, Crassostrea gigas, Octopus bimaculoides and Nematostella vectensis* to perform a TblastN against the transcriptome database of *O. vulgaris*. Nucleotide sequences of the four top hits were retrieved for Pfam analysis (Finn et al., 2006; Finn et al., 2016). Based upon a six-frame translation, protein sequences containing the appropriate number of cadherin (PF00028; https://pfam.xfam.org/family/Cadherin) or cadherin-like domains (PF12733; http://pfam.xfam.org/family/Cadherin-like) were identified as putative protocadherins. Since *de novo* transcriptome assembly is challenging for repeated sequences such as the extracellular region of protocadherins, we chose to include sequences that contain 4,5,6 or 7 extracellular cadherin domains (EC).

To verify sequence identity, a BLASTX against the NCBI non-redundant database was executed (Sayers et al., 2012). When the putative Ov-PCDH sequence matched only with protocadherin sequences in other organisms, we presumed that it was a protocadherin. This resulted in 87 putative protocadherin sequences.

Due to sequence divergence, high stringency of the analysis and transcriptome assembly, a higher number of protocadherins is probable.

O. vulgaris PCDH sequences that were identified to be highly similar were mapped on the *O. bimaculoides* genome using Ensembl Genomes Metazoa Blast (Kersey et al., 2018). When the sequences showed high similarity to the same large exon in the *O. bimaculoides* genome, we concluded that these sequences were part of the same PCDH. This resulted in a total number of 53 putative PCDH.

For comparison, other cadherin transcripts belonging to the cadherin superfamily were identified as well following the same approach. A preliminary analysis of the transcriptome of *O. vulgaris* allowed us to identify several major cadherins (CDH), such as neural cadherin and two CELSR-like sequences. Belonging to the cadherin-related family (CDHR), one dachsous-like, 4 FAT-like, one Ret proto-oncogene-like and one calsyntenin-like genes were present. One FAT-like transcript encodes for 58 extracellular domains.

To identify Ov-DSCAM we used annotated sequences from *Homo sapiens, Mus musculus, Tribolium castaneum, Drosophila melanogaster* and *Crassostrea gigas*. Our annotation was based on the presence of seven immunoglobulin domains (https://pfam.xfam.org/family/PF07679 or https://pfam.xfam.org/family/PF13927), followed by four fibronectin type III domains (PF00041; https://pfam.xfam.org/family/fn3), one immunoglobulin and two other fibronectin domains.

Expression analysis

For the RNA-seq experiments the following nervous tissues were sampled from subadult *O. vulgaris,* obtained from local fishermen (for details see Petrosino, 2015): supra-esophageal mass (SEM), sub-esophageal mass (SUB), optic lobe and the gastric and stellate ganglia. The proximal part of the anterior and posterior arm (L2 and L4, respectively), the arm tip and arm muscle were sampled as well. We analysed the available expression data for the identified DSCAM and PCDH by normalizing the expression values by row and constructing a heatmap using the package ComplexHeatmap in R (Gu et al., 2016). The heatmap utilizes the color palette viridis (Kulesza et al., 2017), where yellow represents high expression and dark blue stands for low expression.

Out of the three biological replicates, expression values were visualised for the animal with an overall greater expression (the tissue sample with the highest total count of CPM).

Phylogenetic reconstruction

We obtained the longest open reading frame of each protein sequence through the translate tool available at ExPASy (Artimo et al., 2012). The presence of the domains in the protein sequence was confirmed again by Pfam (Finn et al., 2016). To study gene evolution a range of other species was included of which the protein sequences were retrieved from UNIPROT (UniProt Consortium 2016) and the appropriate number of domains was verified.

Protein sequences were aligned by MAFFT L-INS-I (v7.037b). We performed 1000 iterations using the Smith-waterman algorithm. Gaps were removed by trimAL (v1.2.rev59). A Bayesian method was used to reconstruct the phylogenetic tree (MrBayes v3.2.6, ngen=6000000, nchains=22). Posterior probability values were added to visualize node significance.

Sequence analysis

We identified the first extracellular cadherin domain (EC1) in sequences of *O. bimaculoides, O. vulgaris, M. musculus* and *H. sapiens* following Hulpiau & Roy (2011). These sequences were aligned in Clustal Omega (Sievers et al., 2011), where the percent identity matrix was calculated. For better visualization, the multiple sequence alignment (ALN format) was then shaded by the BOXSHADE server (RTF format). We used Fuzzpro (Emboss) to search for vertebrate motifs (CM1, CM2, CM3) and octopus-specific motifs in Ov-PCDH protein sequences (see also Albertin et al., 2015).

Moreover, we used the Ov-PCDHs and Ov-DSCAM included in **Figure 2** to assemble multiple sequence alignments (See Sequence Alignments below). *O. vulgaris* sequences (Ov-PCDH6, 28, 50, 52 and Ov-DSCAM) were blasted in Ensembl genome browser and Ensembl Metazoa (Kersey et al., 2018) to retrieve the best match for the following species: *O. bimaculoides*, *L. gigantea*, *C. gigas*, *S. purpuratus*, *D. rerio*, *M. musculus*, *H. sapiens*. For the alignment of Ov-DSCAM we also included *D. melanogaster* in the analysis. Sequences were globally aligned (with free end gaps Blosum62) and trimmed using Geneious 11.1.5 (https://www.geneious.com/). Alignments were shaded by BOXSHADE and protein domains were manually annotated according to the Uniprot protein domain predictions. Percentage identity matrices were calculated by Geneious 11.1.5 and are shown in tables S3-S7.

Results

A total of 53 Ov-PCDH, 17 Ov-CDH and 1 Ov-DSCAM were identified in the transcriptome of *O. vulgaris*. Ov-PCDH and Ov-DSCAM sequences are deposited in GenBank (see **Table S8** for accession numbers).

We found Ov-PCDH transcripts with 4 EC (n=15), 5 EC (n=24), 6 EC (n=10) and 7 EC (n=4). Based on a BLAT against the draft genome of *O. vulgaris,* we concluded that Ov-PCDH genes possess either 5,6 or 7 repeats (data not shown).

By comparing the normalized expression values of these genes, its clearly shown that the PCDHs are differentially expressed throughout the nervous system. Highly expressed PCDH genes (Row Z-score >2) are present in the SEM (n=3), SUB (n=1) and the optic lobes (n=9). There are four genes that are highly expressed in the stellate ganglion and three in the arm tip. Very low expression values (Row Z-score <-1) are only present in gastric ganglion (n=1), the proximal parts of the anterior (n=22) and posterior arms (n=9) and in the arm muscle (n=20). The disparity between nervous and non-nervous tissue is clearly visible in **Figure S1**.

We see an overall lower PCDH expression in the SUB and gastric ganglion compared to other nervous tissues.

Relative expression levels of Ov-PCDH6-C36174G3I1, Ov-PCDH28-C35066G15I1, Ov-PCDH50-C32730G4I1 and Ov-PCDH52-C31207G1I5 and Ov-DSCAM-C34599G6I1[1] are visualized in a representation of the nervous system of *O. vulgaris* (see **Figure 2**, main text).

Due to the absence of expression values for the arm nerve cord, we utilized the expression values of the whole arm tip to represent the expression of the nerve cord in the tip and the expression of the proximal part of the anterior arm to represent the expression of the nerve cord in the arm. The rationale is that the majority of the arm tip consists out of nerve cord while in the arm the nerve cord is much smaller compared to the amount of arm muscle.

[1] GenBank Accession numbers, Protocadherins: MK_216638 (Ov-PCDH6-c36174 g3 i1); MK_216660 (Ov-PCDH28-c35066 g15 i1); MK_216682 (Ov-PCDH50-c32730 g4 i1); MK_216684 (Ov-PCDH52-c31207 g1 i5); GenBank Accession numbers, Dscam: MK_216686 (Ov-Dscam c34599 g6 i1). See also table S8.

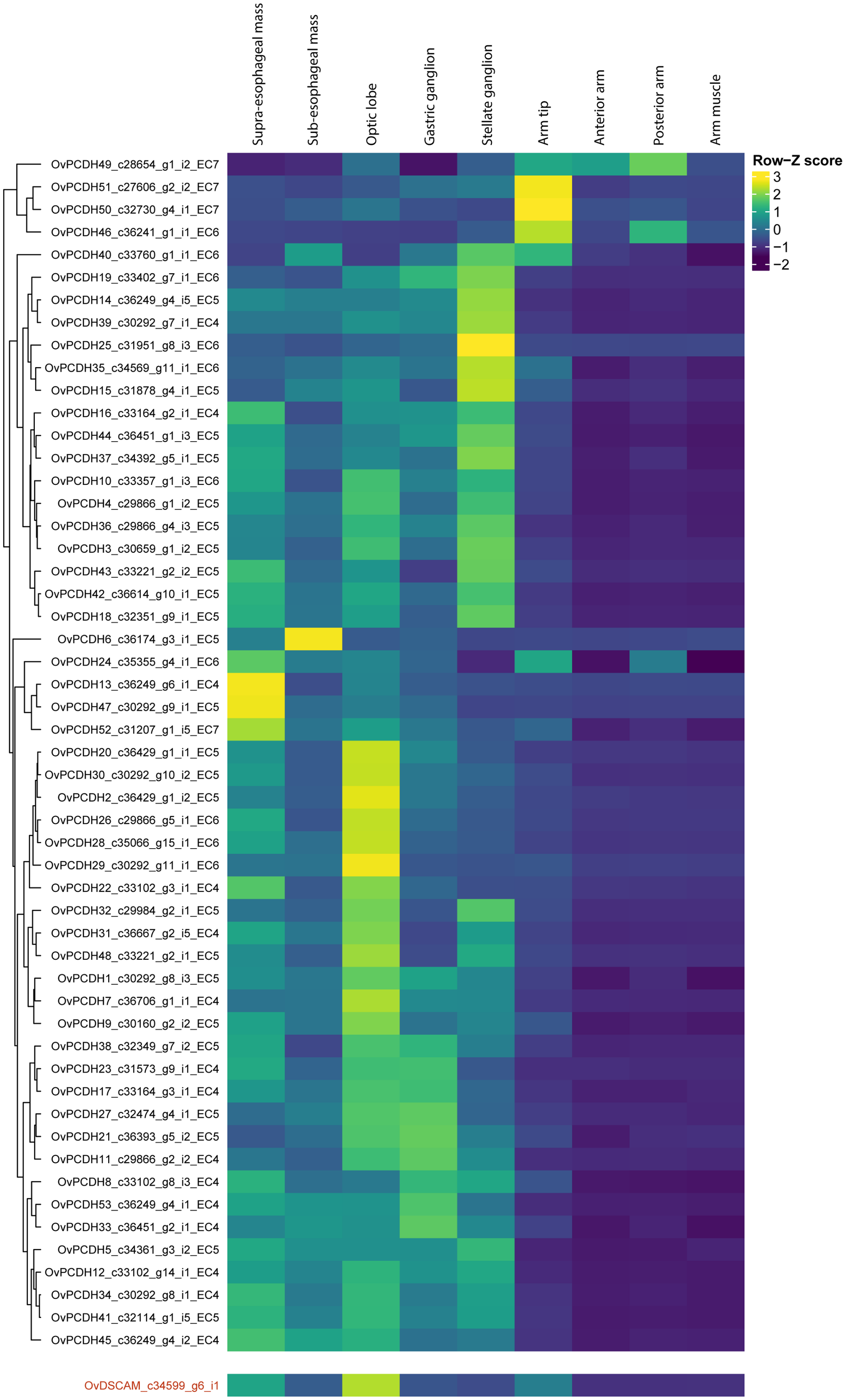

Figure S1: Heatmap of gene expression levels of protocadherins (Ov-PCDH) and Down syndrome cell adhesion molecule (Ov-DSCAM) in *Octopus vulgaris.* Data of expression levels (coded according to Row Z-score) for octopus brain (supra-, sub-esophageal masses and optic lobe), gastric and stellate ganglia, and the arm (proximal parts: anterior and posterior arm; distal part: arm tip; arm muscle only is included) have been based on RNA-seq data (see text for details). Each transcript is uniquely identified and hierarchical clustering based on relative abundance is also provided.

In **Figure S2**, the evolutionary relationships from the PCDH gene family are shown throughout the animal kingdom. PCDHs from invertebrate species (aside from *Branchiostoma floridae* and *Strongylocentrotus purpuratus*) cluster together on the phylogenetic reconstruction. The majority of Ov-PCDH and Ob-PCDH have the same ancestor and are therefore orthologous. Intriguingly, two Ov-PCDH and two Ob-PCDH genes cluster together with other molluscan PCDHs (*Crassostrea gigas*, *Lottia gigantea*, *Biomphalaria glabrata*, *Lingula unguis*). This suggests that these genes are more ancient than the other Ov-PCDH/Ob-PCDH. Coincidently, these genes possess 7 EC (analogous to vertebrate PCDHδ1), which indicates that the other Ov-PCDHs are derived from this Ov-PCDHδ1-like group (visualized in red in **Figure S3**).

In **Figure S3** we also show that the Ov-PCDHδ1-like genes are more cadherin-like than the other Ov-PCDHs since we used an Ov-CDH sequence as an outgroup (visualized in black). Subsequently the same analysis was done to construct the phylogenetic tree of DSCAM genes for a range of different species (isoforms were not included; see **Figure S4**). Ov-DSCAM is clearly molluscan-like and is highly similar to Ob-DSCAM.

As mentioned above, we aligned the first extracellular cadherin domain (EC1; see **Figure S5**) of protein sequences in *O. vulgaris, O. bimaculoides, M. musculus* and *H. sapiens*, following Hulpiau & Roy (2011) and the percent identity matrix was calculated (**Table S1**).

Table S1: Percent Identity Matrix after Clustal2.1

Organism	Percent identity values			
	**(1)**	**(2)**	**(3)**	**(4)**
**(1)** *O. vulgaris*	100	100	33	33
**(2)** *O. bimaculoides*	100	100	33	33
**(3)** *M. musculus*	33	33	100	96
**(4)** *H. sapiens*	33	33	96	100

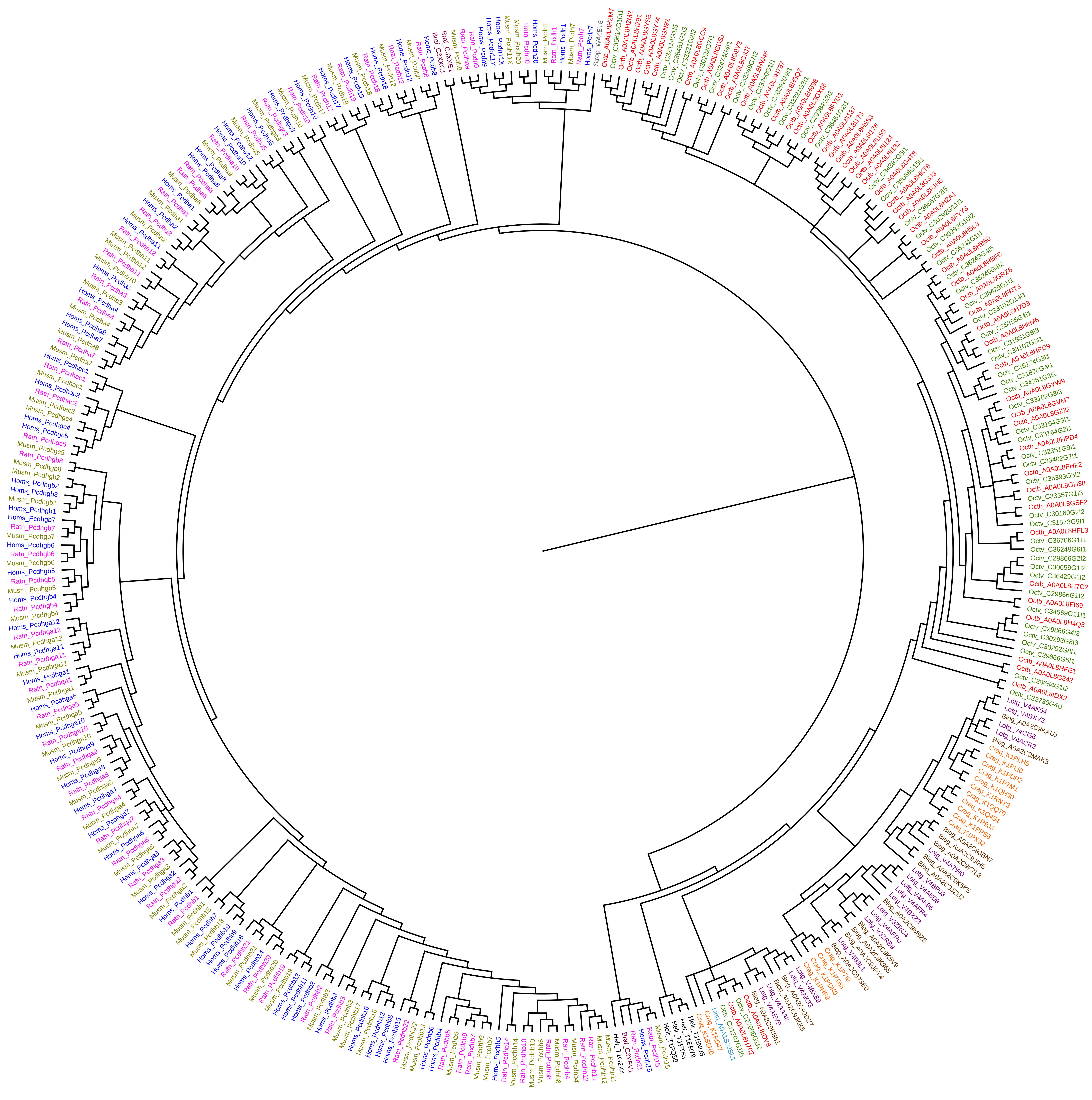

Figure S2: Bayesian phylogenetic reconstruction of the evolutionary relationships between protocadherins in different species. Sequences are color-coded according to species: Homs, *Homo sapiens* (■); Musm, *Mus musculus* (■); Ratn, *Rattus norvegicus* (■); Braf, *Branchiostoma floridae* (■); Strp, *Strongylocentrotus purpuratus* (■); Helr, *Helobdella robusta* (■); Octb, *Octopus bimaculoides* (■); Octv, *Octopus vulgaris* (■); Crag, *Crassostrea gigas* (■); Biog, *Biomphalaria glabrata*(■); Lotg, *Lottia gigantea* (■); Linu, *Lingula unguis* (■). Aside from few exceptions, the invertebrate species are clustered together.

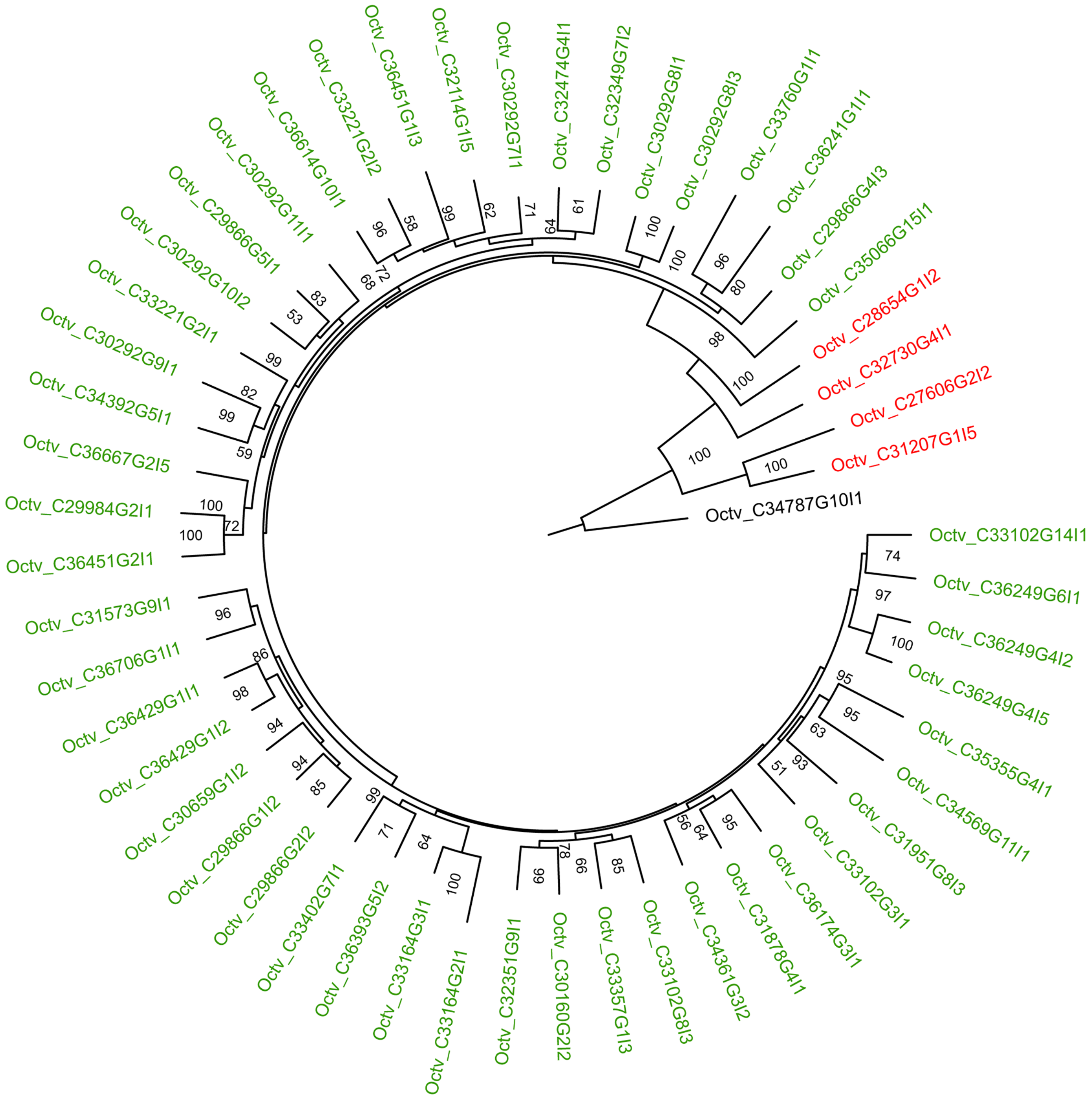

Figure S3: Bayesian phylogenetic tree of Ov-PCDH. Posterior probability values are visualized at each node. Transcripts containing 7 EC are visualized in red. A cadherin gene was used as an outgroup and is shown in black.

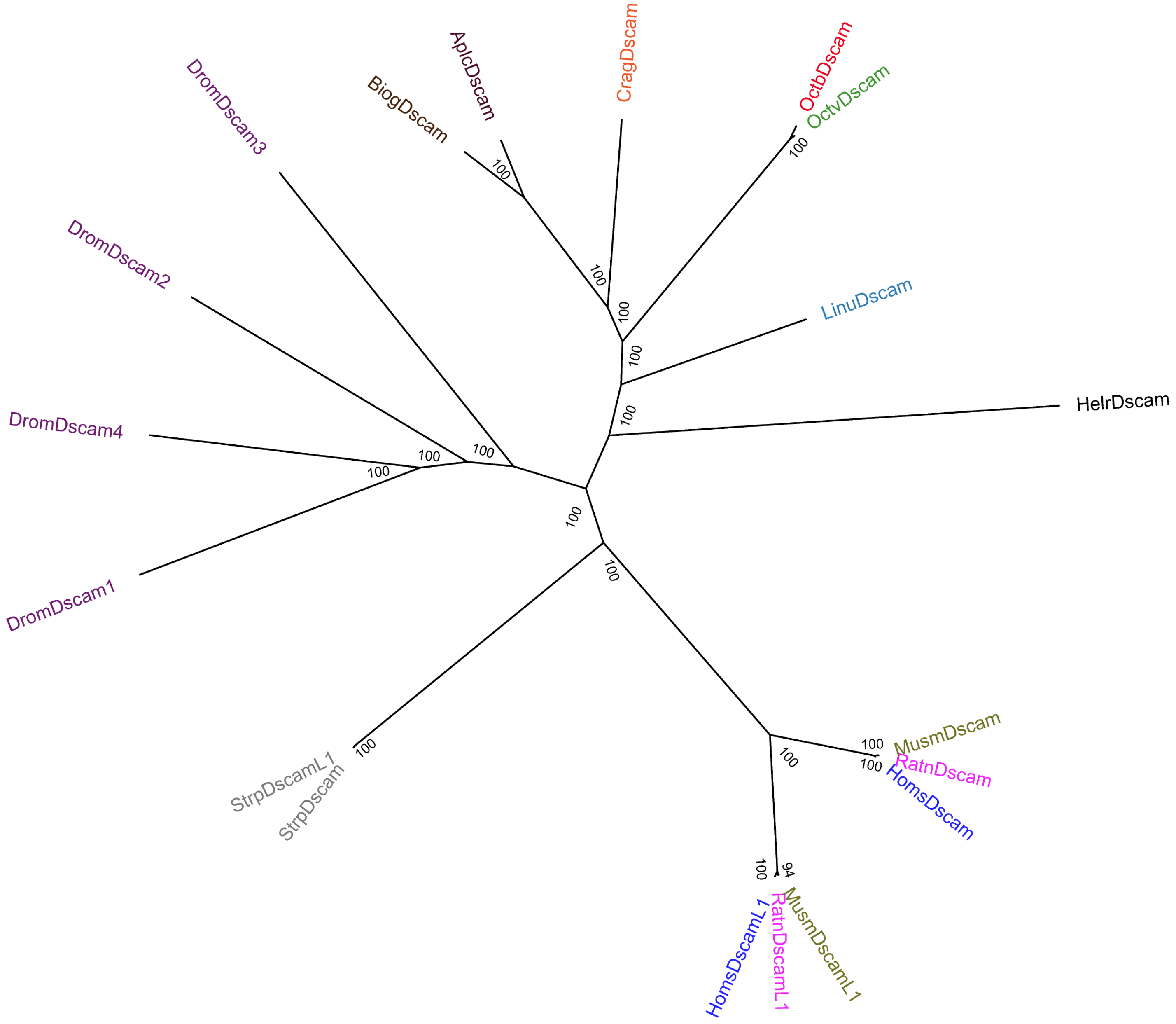

Figure S4: Bayesian phylogenetic reconstruction of the evolutionary relationships between DSCAM in different species. Sequences are color-coded according to species: Homs, *Homo sapiens* (■); Musm, *Mus musculus* (■); Ratn, *Rattus norvegicus* (■); Strp, *Strongylocentrotus purpuratus* (■); Helr, *Helobdella robusta* (■); Octb, *Octopus bimaculoides* (■); Octv, *Octopus vulgaris* (■); Crag, *Crassostrea gigas* (■); Biog, *Biomphalaria glabrata* (■); Drom, *Drosophila melanogaster* (■); Aplc, *Aplysia californica* (■); Linu, *Lingula unguis* (■).

```
O. vulgaris       1  FMNTVQTWNSKNLITFKQLQDSENLDHKKLFNVSKSGKIYTTETLDAETL
O. bimaculoides   1  FMNTVQTWNSKNLITFKQLQDSENLDHKKLFNVSKSGKIYTTETLDAETL
M. musculus       1  --GS--GSGRSKSGSYRVL---ENSAPHLLDVDADSGLLYTKQRIDRESL
H. sapiens        1  --RG--GGGRSKSGSYRVL---ENSAPHLLDVDADSGLLYTKQRIDRESL

O. vulgaris      51  CKYNTECFQIVEVAVRKKQSFIKILEVKIIIIDINDNSP
O. bimaculoides  51  CKYNTECFQIVEVAVRKKQSFIKILEVKIIIIDINDNSP
M. musculus      44  CRHNAKCQLSLEVFANDKE----ICMIKVEIQDINDNAP
H. sapiens       44  CRHNAKCQLSLEVFANDKE----ICMIKVEIQDINDNAP
```

Figure S5: Multiple sequence alignment of EC1. Protein sequences of *O. vulgaris* (c36174_g3_i1, MK_216638), *O. bimaculoides* (Ocbimv22009804), *M. musculus* (ENSMUSG00000035566) and *H. sapiens* (ENSG00000118946) were aligned by Clustal Omega. The motif DXNDXXP (purple) characterizes the cadherin repeat and is present in all sequences.

No vertebrate-like motifs were found in the Ov-PCDHs, but octopus-specific motifs as identified by Albertin et al. (2015) resulted abundant in *O. vulgaris* (**Table S2**).

Table S2: Octopus-specific motifs present in Ov-PCDH*.*

Conserved motifs	PROSITE-style pattern	Reported hitcount
EC1	X(2)[YLF][IVLA][GA][DN][IV]XA[DN]	21
EC1	D[AT]EXXC	45
EC5	[IV][LFS][IVA][KTSIR]D[NCSK]GXPXL	36
EC5	XDXNDN[APVTS]PY	29
EC6	L[RK][AVS][SLVA]D[RKIN]DX[HRG]XN	12
EC6	QNDAG	10
TM	[IV][IV][IVA]XXX[AV][VI]XX[SA]XX	12
PP1	[RK][VI]XF	15

Sequence Alignments

Ov-PCDH6-c36174_g3_i1

```
                                                          EC1
O. vulgaris          1  VREGQKPFTLVGDIAADIQFMNTVQTWNSKNLITFKQLQDSENLDHKK--LFNV-SKSGK
O. bimaculoides      1  VREGQKPFTLVGDIAADIQFMNTVQTWNSKNLITFKQLQDSENLDHKK--LFNV-SKSGK
L. gigantea          1  LMESKPSGTLVGNIAAETNIARGISISG----FKSLRYSFLNPNDVDIASLFSVDSTTSD
C. gigas             1  LLEQQSRETFVGNVAVDSLLKANVTQEE----LERMKFQIL-TQGSKDASYFIIDEKSST
S. purpuratus        1  IDEGVGPGTVIGNVADDIAITIDA------------NTEFSMLGVPNETAYVSLDSQTGE
D. rerio             1  ISEEADPGTTVGPIAKDLNLNLH-----------ELQLRGFQLVSGPNKRYFDVNLKSGV
M. musculus          1  VPEEQGAGTVIGNIGKDARLQPGLPPAERGSGSGRSKSGSYRVLENSAPHLLDVDADSGL
H. sapiens           1  IYEEQRVGSVIARLSEDVADVLLKLP--------NPSTVRFRAMQRGNSPLLVVNEDNGE

O. vulgaris         58  IYTTETLDAETLC-KYNTECFQIVEVAVRK-----KQS-FIKILEVKIIIIDINDNSPEF
O. bimaculoides     58  IYTTETLDAETLC-KYNTECFQIVEVAVRK-----KQS-FIKILEVKIIIIDINDNSPEF
L. gigantea         57  ITSNKLIDREKVC-EFTADCVLTFDVKINS-----LLTSFFEIVTIKIIVDDVNDNAPIF
C. gigas            56  IKTASVLDREVLC-EFEVKCVLEFSVAVYKQDQQHSSLDLFKIFAIKVNILDANDNAPTF
S. purpuratus       49  LTTVLDLDREELCPGSSALCEIEVNAIELGT---------REVITVKVTINDINDHAPEF
D. rerio            50  LLVKERIDRELLC-GRSSRCSLEIEAIVNSP---------LNMYRLEVNVLDINDNGPIF
M. musculus         61  LYTKQRIDRESLC-RHNAKCQLSLEVFAND----------KEICMIKVEIQDINDNAPSF
H. sapiens          53  ISIGATIDREQLC-QKNLNCSIEFDVITLPT-------EHLQLFHIEVEVLDINDNSPQF

                        EC2
O. vulgaris        111  PFRKVRLEFYETDGKNTTKSIPNAFDRDVGLLNSKIVYHLKKHIDEPFSLSTSKRVVGNS
O. bimaculoides    111  PFRKVRLEFYETDGKNTTKSIPNAFDRDVGLLNSKIVYHLKKHMDEPFSLSTSKRVVGNS
L. gigantea        111  PESEITVFIPENVNPGTMYRIDGATDKDRGKNNSVQSYEMISSAN-TFGLKVDKKLDGTS
C. gigas           115  PQSQVALDVQESVPVDFVLLTSGAVDPDMGINNSIKSYTLKPS-NEMFGLKEIKNIDGTT
S. purpuratus      100  RDDLTNMSIPESVVPGTRFPLSTASDEDIG-ENAIQGYRLSDEYAETFGLVQNEFPGGLI
D. rerio           100  KSSKTELNIVESAFPGERFTLPDAFDADVG-SNSVKSYKLSA--NEHFTLDVQSGGEQSV
M. musculus        110  PSDQIEMDISENAAPGTRFPLTSAHDPDAG-ENGLRTYLLTRDDHGLFALDVKSRGDGTK
H. sapiens         105  SRSLIPIEISESAAVGTRIPLDSAFDPDVG-ENSLHTYSLSA--NDFFNIEVRTRTDGAK

                                                                           EC3
O. vulgaris        171  KLVIILQGKLDREMKDSYSLQIIAKDSGTPSKQDVLDVEITVTDENDNAPVFSQNIYNVS
O. bimaculoides    171  KLVIILQGKLDREMKDSYSLQIIAKDSGTPSKQDVLNVEITVTDENDNAPVFSQNIYNVS
L. gigantea        170  DVRIIVKNVIDREKKNYYRFFIIAKDGGNPPLSGNVTVNVNVSDENDNAPEFSEQHYDVS
C. gigas           174  DLGLVVRYKLDRETLDFYQVEIVAKDGGFPQRSGTVMVNITVIDDNDNKPLFSQAKYDAS
S. purpuratus      159  IIQLEVIGSLDRENKDNYVMTLYADDGGDPVLSGVTTLNVTVLDSDDHSPVFDRTSYQVS
D. rerio           157  SAELVLQKALDREKQPVIKLTLTAVDGGKPQKSGTTQIIINVEDVNDNIPVFSTSLYKTR
M. musculus        169  FPELVIQKALDRELQNHHTLVLTALDGGEPPRSATVQINVKVIDSNDNSPVFEAPSYLVE
H. sapiens         162  YAELIVVRELDRELKSSYELQLTASDMGVPQRSGSSILKISISDSNDNSPAFEQQSYIIQ

O. vulgaris        231  VNKAHQIGKPAVILSTKDLDLGKNAEVTYHFDSKTSVAVKNFFKLNSETGEIFLSKNFPL
O. bimaculoides    231  VNKAHQIGKPAVILSTKDLDLDKNAKVTYHFDSKTSVAVKNFFKLNSETGEIFLSKNFPL
L. gigantea        230  VTENTPVYSVIAKIHATDRDSGMNAKVVYRFSPHKSPEIEKLFALNPENGDISVRNELQY
C. gigas           234  IPENHPVGKNVLTLSAQDLDINENGEFTFAFNSRVPQKIKDKFAVNKTSGEIYTISEIDY
S. purpuratus      219  VAENIGVGQHIIQVRASDPDTGTNGQIIYDFGGSVSAKIIELFEIDSESGWLSVKSELDF
D. rerio           217  IMENAAVGTSVITVQASDADEGLNGEIIYSFISHDGDNRVNAFTIDSVSGVISVKGNIDY
M. musculus        229  LPENAPLGTVVIDLNATDADEGPNGEVLYSFSSYVPDRVRELFSIDPKTGLIRVKGNLDY
H. sapiens         222  LLENSPVGTLLLDLNATDPDEGANGKIVYSFSSHVSPKIMETFKIDSERGHLTLFKQVDY

                                                                     EC4
O. vulgaris        291  DKRQTYKLFIDATDSGNPPLSSTAIVLINVLNQQNNAPVIDVNFVSDSKGAMLTISEGVE
O. bimaculoides    291  DKRQTYKLFIDATDSGNPPLSSTAIVLINVLNQQNNAPVIDVNFVSDSKGTMLTISEGIE
L. gigantea        290  QSGNQFETIIEASDQGVPEQVSQAKLTLRILDVGNNPPLVTINPVSSGVGDMVLISEGAR
C. gigas           294  EEEDNYQFLVEVQDKGREPKSSTSVVNIIILDVNDNAPQISVNLLPDG----TDILESAE
S. purpuratus      279  EDESSHQVSIRATNNVPNPLPDFTTVTVNLIDVNDNKPRLTISALGDG-GRFKHIAENSP
D. rerio           277  ETSNAVEIRVQAKDKGQKPRAAHCKVLIEIVDVNDNIPEISVTSLAE------TVREDAA
M. musculus        289  EENGMLEIDVQARDLGPNPIPAHCKVTVKLIDRNDNAPSIGFVSVRQG-----ALSEAAP
H. sapiens         282  EITKSYEIDVQAQDLGPNSIPAHCKIIIKVVDVNDNKPEININLMSPGKEEISYIFEGDP

O. vulgaris        351  VGSFIAYIKVTDKDAGRNGEVTCNLRHDK-L------------QLKSLGRGKFKVVVKSP
O. bimaculoides    351  VGSFIAYIKVTDKDAGRNGEVTCSLRHDK-L------------QLKSLGRGKFKVVVKSP
L. gigantea        350  VGTVVAHVNIVDKDVGPNGQVQCNCPHDF-F------------SVHRLEGRGYIVQVKRI
C. gigas           350  VGRYVANFAVSDLDSGPNGEIQCQVLGEF-------------FKIEEIFNNMYKVIIKSP
S. purpuratus      338  EDVDVAYVRVTDMDIGVNGQAILTLEDDFG-H----------FYLESFREGQYFLKTAGV
D. rerio           331  AGTMVGLITVKDGDAGKNGAVVLNIRGSA-P-----------FRIQNTYKNKYYLLVDGQ
M. musculus        344  PGTVIALVRVTDRDSGKNGQLQCRVLGGGGTGGGLGGPGSVPFKLEENYDNFYTVVTDRP
```

```
                                                                                      EC5
H. sapiens           342  IDTFVALVRVQDKDSGLNGEIVCKLHGHGH------------FKLQKTYENNYLILTNAT
O. vulgaris          398  VDRETEKRIDINISCRDNGSPSLMTERKFTIEVNDVNDVKPQFTKTIFRFLTYENEEPNF
O. bimaculoides      398  VDRETEKRIDINISCRDNGTPSLMTERKFTIEVNDVNDVKPQFTKTIFRFLTYENEEPNF
L. gigantea          397  LDRERNDEHKVMVICRDSGTPRLSARAKFTVRLTDMNDEPPVFTKRVFETSVEENNVIGR
C. gigas             397  LDYESRHVHNVTIQCQDQGIPQHQNTSSFLINVLDVNDNNPVFLQSIYRATIKENNPPNE
S. purpuratus        387  LDREDIDFYNITILAEDRGSPVLSSRRFAVFVDDENDNSPIFSSSVYHATISENNEPGH
D. rerio             379  LDRETASEYNVTISAADEGSPPLSSTSVIAVHVSDVNDNAPRFPEPVINVYVKENSQIGA
M. musculus          404  LDRETQDEYNVTIVARDGGSPPLNSTKSFAVKILDENDNPPRFTKGLYVLQVHENNIPGE
H. sapiens           390  LDREKRSEYSLTVIAEDRGTPSLSTVKHFTVQINDINDNPPHFQRSRYEFVISENNSPGA

O. vulgaris          458  PVGFINATDPDLGPGGQLSYSLLTKMKDALPFEI-----SNYGFISTTKSLDREKQDLYK
O. bimaculoides      458  PVGFINATDPDLGPGGQLSYSLLTKMKDALPFEI-----SNYGFISTTKSLDREKQDLYK
L. gigantea          457  PIIRISANDADIGKNAIVQYHLNLADDSMFRIN------QNTGEIVANARFDRETMSEMK
C. gigas             457  VITTVKAVDKDSGLAGKVIYFMHTDGSDSFHVD------STSGVVTVKKSLDREISPVIL
S. purpuratus        447  RVATVQAIDKDELENGEVVYSLLDDKDGSFGIH------PFNGVLTANVSLDREDGESID
D. rerio             439  VLHTVSAVDPDVGDNARITYSLLESSKSG-PVTSMININSDTGDLHSLQSFNYEEIKTFE
M. musculus          464  YLGSVLAQDPDLGQNGTVSYSILPSHIGDVSIYTYVSVNPTNGAIYALRSFNYEQTKAFE
H. sapiens           450  YITTVTATDPDLGENGQVTYTILESFILGSSITTYVTIDPSNGAIYALRIFDHEEVSQIT
                                                                                EC6
O. vulgaris          513  FKVLVRDNGTPS-LNNTANVVVEV------------------------------------
O. bimaculoides      513  FKVLVKDNGTPS-LNNTANVVVEVMDENDNAPYFTFPSVNPFKLS---VQYNPESPSDIT
L. gigantea          511  FTVRAVDEGNPP-LTGTAEVLVRIIDENDNRPTFDASSLVFS------INEDKKPGSEVG
C. gigas             511  FHVNASDAGNPQ-LKSSTLIRITLEDENDNTPMFKKSHFEFY------VLEEQKNLPIVG
S. purpuratus        501  LMIRACDRGQPQGC-SDVPLTVRVLDMNDNGPTFGGDLIEMR------IDENKPSGTIVG
D. rerio             498  FKVQATDSGVPP-LSSNVTVNVFVLDENDNSPAILAPYSELGSVNTENIPYSAEAGYFVA
M. musculus          524  FKVLAKDSGAPAHLESNATVRVTVLDVNDNAPVIVLPTLQNDTAELQ-VPRNAGLGYLVS
H. sapiens           510  FVVEARDGGSPKQLVSNTTVVLTIIDENDNVPVVIGPALRNNTAEIT-IPKGAESGFHVT

O. vulgaris               ------------------------------------------------------------
O. bimaculoides      569  ILKAMDKDIRENAFLRYEILGGNENRLFTLTPYTGVLFFSRSIKPKDSNLYRLLIAVKDS
L. gigantea          564  ILRAKDADFNFNSDVEYAMIVNGAENVPFAVFSNGVIRTNKALDYEEQKRYSFNIIAKDK
C. gigas             564  RLFAEDPDAGPNGQFSFDFASPRYPE-FILDYDTGLLKAGM-LDRELKTVYNFNVTVTDK
S. purpuratus        554  RAVATDADEGDNGRLRYSILT---DAVFRIDEDSGRIYSTAELDREIQELYHFTVRAVDD
D. rerio             557  KIRAVDADSGYNALLSYHISEPKGNNLFRIGTSSGEIRTKRRMSDNDLKTHPLVILVCDN
M. musculus          583  TVRALDSDFGESGRLTYEIVDGNDDHLFEIDPSSGEIRTLHPFWEDVTPVVELVVKVTDH
H. sapiens           569  RIRAIDRDSGVNAELSCAIVAGNEENIFIIDPRSCDIHTNVSMDSVPYTEWELSVIIQDK
                                                                              TM
O. vulgaris               ------------------------------------------------------------
O. bimaculoides      629  GAVVLSATTTLSLRLTATNKTMGKTSNTNTISDSEISIKLGVIIVVAAVTISLTIIVSIA
L. gigantea          624  GRPPL--NTTARVLVYVMDANDHSPIIVFPNSHNDTITV----TSDTEPGTVITKVVAKD
C. gigas             622  GNPPRSSMALVTVHVL--DANDNMPRIIYPDNHNNTIK----LMYTTPKDSVIARVEADD
S. purpuratus        611  GLSPKTATATVVVTV--NDCNDHSPEFIVPSAKNDIRF----IPVSADPGLHIMTVESED
D. rerio             617  GEPSLSATVSIDAVVVESGGDVKTPFRHAPVKEESFSDLNLYLLIAIVSVSVIFLLSLIS
M. musculus          643  GKPTLSAVAKLIIRSVSGSLPEGVPR-VNGEQHHWDMSLPLIVTLSTISIILLAAMITIA
H. sapiens           629  GNPQLHTKVLLKCMIFEYAESVTSTAMTSVSQASLDVSMIIIISLGAICAVLLVIMVLFA

O. vulgaris               ------------------------------------------------------------
O. bimaculoides      689  MCMVQNNPQRNIYYNRSASANNTIWGHTTKAECFCDQIPSHYDSPSTKTTIVPRNDSYHL
L. gigantea          678  RDEGDNARLSYYINKGNVDNTFHI--GSKSGEIVLARRLMLDEREDYHLTVSVQDQGKTQ
C. gigas             676  IDEGNNSVLSFYIHKVEPTKPDLFKMNAETGELMIAKTMHLYDSDSYRLVLGVKNGVFTM
S. purpuratus        665  EDKDENAAVSYAISHGNTNGAF----GIQANGQVVTAQDLEPMWEGVHDITIRATDGGNP
D. rerio             677  LIAVKCHRTDSSLGRYSAPMITTHPDGSWSYSKSTQQYDVCFSSDTLKSDVVVFP---AP
M. musculus          702  VKCKRENKEIRTYNCRIAEYSHPQLGGGKGKKKKINKNDIMLVQSEVEERNAMNVMNVVS
H. sapiens           689  TRCNREKKDTRSYNCRVAESTYQH--HPKRPSRQIHKGDITLVPTINGTLPIRSHHRSSP

O. vulgaris               ------------------------------------------------------------
O. bimaculoides      749  MKFRREPCSRYLTNSYNRKDLSGFPCQATTEVCSQNDHGSDKEAAICAPYSEISLMYHTD
L. gigantea          736  QASQTYLTVRIIYVNVTYIDSTHEDLRYIIIAGVVTGVTVILSIIIVAVILYVRRSDNQR
C. gigas             736  FA-NLNVIITVSNNTVLGAQSPGSGENNIVIVIAIVVVTVILSVAIIAAICIVKYVDRHR
S. purpuratus        721  SASSTAVLRVVIANEGFKRSLPPANFTALFNLTIDYYLNLGNNAASSRGILNDWPMIVII
D. rerio             734  FPPADAELISINGGDTFTRTQTLPNKEKPKVPSSDWRYSASLRAGMQSSVRMEESSVMQG
M. musculus          762  SPSLATSPMYFDYQTRLPLSSPRSEVMYLKPASNNLTVPQGHAGCHTSFTGQGTNSSETP
H. sapiens           747  SSSPTLERGQMGSRQSHNSHQSLNSLVTISSNHVPENFSLELTHA-TPAVEQVSQLLSML
```

Table S3: Percentage identity matrix of Ov-PCDH6.

Ov-PCDH6	Accession number	*Ov*	*Ob*	*Lg*	*Cg*	*Sp*	*Dr*	*Mm*	*Hs*
O. vulgaris	c36174_g3_i1[2]	100	98	32	33	27	29	29	28
O. bimaculoides	Ocbimv22009804	98	100	26	26	22	24	24	24
L. gigantea	LotgiT51526	32	26	100	32	29	25	26	25
C. gigas	EKC42706	33	26	32	100	28	26	27	25
S. purpuratus	SPU_025622	27	22	29	28	100	27	28	28
D. rerio	ENSDART00000124485	29	24	25	26	27	100	35	32
M. musculus	ENSMUSG00000035566	29	24	26	27	28	35	100	38
H. sapiens	ENST00000344876	28	24	25	25	28	32	38	100

[2] For the corresponding GenBank Accession Number refer to table S8.

Ov-PCDH28-c35066_g15_i1

```
                   EC1
O. vulgaris       1 VEEEKSPGTYVGDIAADTQLLDSIPVENPDLIRFSQLQQS----ATSDSDLFNVS-RTGK
O. bimaculoides   1 VEEEKSPGTYVGDIAADTQLLDSIPVENPELIRFSQLQQS----ATSDSDLFNVS-RTGK
L. gigantea       1 LMESKPSGTLVGNIAAETNIARGISISGFKSLRYSFLNPN----DVDIASLFSVDSTTSD
C. gigas          1 LLEQQSRETFVGNVAVDSLLKANVTQEELERMKFQILTQ-----GSKDASYFIIDEKSST
S. purpuratus     1 IDEGVGPGTVIGNVADD------LAITIDANTEFSMLG------VPNETAYVSLDSQTGE
D. rerio          1 LPEEMKRGSVIGNIAKDL----GLDVNRLSSRKARIDT------EGNRKRYCDINLNTGE
M. musculus       1 VPEEQGAGTVIGNIGKDARLQPGLPPAERGSGSGRSKSGSYRVLENSAPHLLDVDADSGL
H. sapiens        1 VPEETDKGSFVGNIAKDL----GLQPQELADGGVRIVS-------RGRMPLFALNPRSGS

O. vulgaris      56 LYTAKVLDAETLC-IYNVECFKTIKIAV------HQAGTFMRILKIKVFIKDVNDHEPKF
O. bimaculoides  56 LYTAKVLDAETLC-VYNVECFKTIKIAV------HQAGTFMRILKIKVFIKDVNDHEPKF
L. gigantea      57 ITSNKLIDREKVC-EFTADCVLTFDVKI----NS-LLTSFFEIVTIKIIVDDVNDNAPIF
C. gigas         56 IKTASVLDREVLC-EFEVKCVLEFSVAVYKQDQQHSSLDLFKIFAIKVNILDANDNAPTF
S. purpuratus    49 LTTVLDLDREELCPGSSALCEIEVNAIE-L---------GTREVITVKVTINDINDHAPEF
D. rerio         51 LTVAERIDREGLC-GKKSSCVLNQELVL----------ENPLELHRIGLRVQDINDNNPYF
M. musculus      61 LYTKQRIDRESLC-RHNAKCQLSLEVFA----------NDKEICMIKVEIQDINDNAPSF
H. sapiens       50 LITARRIDREELC-AQSMPCLVSFNILV----------EDKMKLFPVEVEIIDINDNTPQF
                   EC2
O. vulgaris     109 PDKQIELFFDENDKEGTSQSIPDAVDKDVGILNSQITYQLRKNSDEPFTLSTSKRVDGRA
O. bimaculoides 109 PDKEVELFFDENDKEGTTQSIPDAVDKDVGILNSQITYQLRKNSDEPFTLSTSKRVDGRA
L. gigantea     111 PESEITVFIPENVNPGTMYRIDGATDKDRGKNNSVQSYEMISSAN-TFGLKVDKKLDGTS
C. gigas        115 PQSQVALDVQESVPVDFVLLTSGAVDPDMGINNSIKSYTL-KPSNEMFGLKEIKNIDGTT
S. purpuratus   100 RDDLTNMSIPESVVPGTRFPLSTASDEDIG-ENAIQGYRLSDEYAETFGLVQNEFPGGLI
D. rerio        101 GKDLINLEISESAVKGKRFLLEEANDADIG-QNSIQSYSIQNNEY--FILSMQANSFEEK
M. musculus     110 PSDQIEMDISENAAPGTRFPLTSAHDPDAG-ENGLRTYLLTRDDHGLFALDVKSRGDGTK
H. sapiens      100 QLEELEFKMNEITTPGTRVSLPFGQDLDVG-MNSLQSYQLSSNPH--FSLDVQQGADGPQ
                                                                        EC3
O. vulgaris     169 KLEITLEAKLDRELRDNYMVQIVSKDGGFPSKEGLLNVKISVNDENDNPPVFSQSIYNVS
O. bimaculoides 169 KLEITLEAKLDRELRENYLVQIVSKDGGFPSKQGLLNVKISVNDENDNPPVFSQSIYNIS
L. gigantea     170 DVRIIVKNVIDREKKNYYRFFIIAKDGGNPPLSGNVTVNVNVSDENDNAPEFSEQHYDVS
C. gigas        174 DLGLVVRYKLDRETLDFYQVEIVAKDGGFPQRSGTVMVNITVIDDNDNKPLFSQAKYDAS
S. purpuratus   159 IIQLEVIGSLDRENKDNYVMTLYADDGGDPVLSGVTTLNVTVLDSDDHSPVFDRTSYQVS
D. rerio        158 YAELVLNKELDREKEKEVTLILTAVDGGTPPRSGTVAIHVTVLDANDNAPVFSQAVYKVS
M. musculus     169 FPELVIQKALDRELQNHHTLVLTALDGGEPPRSATVQINVKVIDSNDNSPVFEAPSYLVE
H. sapiens      157 HPEMVLQSPLDREEEAVHHLILTASDGGEPVRSGTLRIYIQVVDANDNPPAFTQAQYHIN

O. vulgaris     229 IKHTHQMNTPVAVLSSKDLDSGRYGRVSYHFSSKTTDLAQSYFQVEENTGEIFAIKQFPS
O. bimaculoides 229 IKHTHQMNTPVAVLSSKDLDSGRYGRVTYHFSSKTTDIAQSYFQVEENTGEIFAIKQFPS
L. gigantea     230 VTENTPVYSVIAKIHATDRDSGMNAKVVYRFSPHKSPEIEKLFALNPENGDISVRNELQY
C. gigas        234 IPENHPVGKNVLTLSAQDLDINENGEFTFAFNSRVPQKIKDKFAVNKTSGEIYTISEIDY
S. purpuratus   219 VAENIGVGQHIIQVRASDPDTGTNGQIIYDFGGSVSAKIIELFEIDSESGWLSVKSELDF
D. rerio        218 LPENSPVDTVVVTVSATDADEGQNGEVTYEFG-HIMEDYKHLFNLDRKTGVISIKGPVDF
M. musculus     229 LPENAPLGTVVIDLNATDADEGPNGEVLYSFSSYVPDRVRELFSIDPKTGLIRVKGNLDY
H. sapiens      217 VPENVPLGTQLLMVNATDPDEGANGEVTYSFH-NVDHRVAQIFRLDSYTGEISNKEPLDF
                                                         EC4
O. vulgaris     289 IQKLSYKLFVDAQDGGTP-PLRSTAIVLITVTNQQNNPPNIDVNFVSAFSENTVTISEGI
O. bimaculoides 289 IQKLSYKLFVDAQDGGNP-PLRSTAIVLITVTNQQNNPPNIDVNFVSAFSENTVTISEGI
L. gigantea     290 QSGNQFETIIEASDQGVPEQV-SQAKLTLRILDVGNNPPLVTINPVSSGVGDMVLLSEGA
C. gigas        294 EEEDNYQFLVEVQDKGRE-PKSSTSVVNIIILDVNDNAPQISVNLLP----DGTDILESA
S. purpuratus   279 EDESSHQVSIRATNNV-PNPLPDFTTVTVNLIDVNDNKPRLTISALGDG-GRFKHIAENS
D. rerio        277 EEEATFSLRIIAKDGS---GLTSYSNVLISVSDVNDNSPIIIVKSL------NIPIPESA
M. musculus     289 EENGMLEIDVQARDLG-PNPIPAHCKVTVKLIDRNDNAPSIGFVSV-----RQGALSEAA
H. sapiens      276 EEYKMYSMEVQAQDGA---GLMAKVKVLIKVLDVNDNAPEVTITSVT------TAVPENF

O. vulgaris     348 KVGSFIAYVMVTDNDIGRNGEVTCSL-D------------HDRFQLRTMETKEYKVLLKN
O. bimaculoides 348 KVGSFIAYVMVTDNDIGRNGEVTCSL-D------------HDRFQLRSMEVKEYKVLLKN
L. gigantea     349 RVGTVVAHVNIVDKDVGPNGQVQCNC-P------------HDFFSVHRLEGRGYIVQVKR
C. gigas        349 EVGRYVANFAVSDLDSGPNGEIQCQVL----------G---EFFKIEEIFNNMYKVIIKS
S. purpuratus   337 PEDVDVAYVRVTDMDTGVNGQAILTLE-----------DDFGHFYLESFREGQYFLKTAG
D. rerio        328 LPGTEVGIINVQDRDSENNGQVRCSIQQ------------NVPFKLVPSIKNYYSLVTTG
M. musculus     343 PPGTVIALVRVTDRDSGKNGQLQCRVLGGGGTGGGLGGPGSVPFKLEENYDNFYTVVTDR
H. sapiens      327 PPGTIIALISVHDQDSGDNGYTTCFIPG------------NLPFKLEKLVDNYYRLVTER
```

```
                                                                            EC5
O. vulgaris        395 SVDREAKNLFNVRIICQDRGHPPLQTEKKFFIKVVDVNDVQPQFTKKTFKFLTYENEEVN
O. bimaculoides    395 SVDREAKNLFNVKIICQDRGHPPLQTEKKFFIKVVDVNDVQPQFTKKTFKFLTYENEEVN
L. gigantea        396 ILDRERNDEHKVMVICRDSGTPRLSARAKFTVRLTDMNDEPPVFTKRVFETSVEENNVIG
C. gigas           396 PLDYESRHVHNVTIQCQDQGIPQHQNTSSFLINVLDVNDNNPVFLQSIYRATIKENNPPN
S. purpuratus      386 VLDREDIDFYNITILAEDRGSPVLSSRRRFAVFVDDENDNSPIFSSSVYHATISENNEPG
D. rerio           376 ELDRELLSEYNITITATDEGSPPLSSTKNIHLTVADVNDNPPVFQQQNYRAHVQENNKAG
M. musculus        403 PLDRETQDEYNVTIVARDGGSPPLNSTKSFAVKILDENDNPPRFTKGLYVLQVHENNIPG
H. sapiens         375 TLDRELISGYNITITAIDQGTPALSTETHISLLVTDINDNSPVFHQDSYSAYIPENNPRG

O. vulgaris        455 FPVGLVNATDPDMGAGGQLTYSLYGKNATLLPFKI-----TDNGFILTKRALDYERQDIY
O. bimaculoides    455 FPVGLVNATDPDMGAGGQLTYSLYGKNATLLPFKI-----TDNGFILTKRALDYERQDIY
L. gigantea        456 RPIIRISANDADIGKNAIVQYHLNLADDSMFRIN------QNTGEIVANARFDRETMSEM
C. gigas           456 EVITTVKAVDKDSGLAGKVTYFMHTDGSDSFHVD------STSGVVTVKKSLDREISPVI
S. purpuratus      446 HRVATVQAIDKDELENGEVVYSLLDDKDGSFGIH------PFNGVLTANVSLDREDGESI
D. rerio           436 SSICSVSATDPDWRQNGTVVYSLLSSDVNGAPVSSFLSINGDTGVIHAVRSFDYEQMKSF
M. musculus        463 EYLGSVLAQDPDLGQNGTVSYSILPSHIGDVSIYTYVSVNPTNGAIYALRSFNYEQTKAF
H. sapiens         435 ASIFSVRAHDLDSNENAQITYSLIEDTIQGAPLSAYLSINSDTGVLYALRSFDYEQFRDM

                                                                     EC6
O. vulgaris        510 VFQVLVKDNGIPP-LNNTVNVVVEVMDENDNAPYFTFPSI--S-PFNLDV-YYEPHNNKK
O. bimaculoides    510 VFQVLVKDNGIPP-LNNTVNVVVEVMDENDNAPYFTFPSI--S-PFNLDV-YYEPHNNKK
L. gigantea        510 KFTVRAVDEGNPP-LTGTAEVLVRIIDENDNRPTFDA-----S-SLVFSI-NEDKKPGSE
C. gigas           510 LFHVNASDAGNPQ-LKSSTLIRLTLEDENDNTPMFKK-----S-HFEFYV-LEEQKNLPI
S. purpuratus      500 DLMIRACDRGQPQ-GCSDVPLTVRVLDMNDNGPTFGG-----D-LIEMRI-DENKPSGTI
D. rerio           496 KVLVLARDNGSPP-LSSNVTVSVFISDENDNSPQILYPSPEGN-SFMTEMVPKAAQARSL
M. musculus        523 EFKVLAKDSGAPAHLESNATVRVTVLDVNDNAPVIVLPTLQND-TAELQV-PRNAGLGYL
H. sapiens         495 QLKVMARDSGDPP-LSSNVSLSLFLLDQNDNAPEILYPALPTDGSTGVELAPLSAEPGYL

O. vulgaris        565 IITLKAIDNDSPRNSFLRYKIIRGNNKQLFTINPYTGVLTFSRTVYQSDAGLYYLQCIVK
O. bimaculoides    565 IITLKAIDNDSPRNSFLRYKIIRGNNKQLFTINPYTGVLTFSRTVYQSDAGLYYLQCIVK
L. gigantea        562 VGILRAKDADFNFNSDVEYAMLVNGAENVPFAVFSNGVIRTNKALDYEEQKRYSFNIIAK
C. gigas           562 VGRLFAEDPDAGPNGQFSFDFASPRYPE-FILDYDTGLLKAGM-LDRELKTVYNFNVTVT
S. purpuratus      552 VGRAVATDADEGDNGRLRYSILT---DAVFRIDEDSGRIYSTAELDREIQELYHFTVRAV
D. rerio           554 VSKVIAVDADSGQNAWLSYHIIKATDPGLFTIGVHSGEIRTQRDISESDSMKQNLIVSVR
M. musculus        581 VSTVRALDSDFGESGRLTYEIVDGNDDHLFEIDPSSGEIRTLHPFWEDVTPVVELVVKVT
H. sapiens         554 VTKVVAVDRDSGQNAWLSYRLLKASEPGLFSVGLHTGEVRTARALLDRDALKQSLVVAVQ

                                                                          TM
O. vulgaris        625 DSGIPVLSAASNLSITLTVSNKTSEKMAVAHS----DSDKRIHLSLVVIITLAAVTVSMA
O. bimaculoides    625 DSGIPVLSAASNLSITLTVSNKTSEKMAVAHS----DSDKRIHLSLVVIITLAAVTVSMA
L. gigantea        622 DKGRPPLNTTARVLVYVMDANDHSPIIVFPNSHNDTITVTSDTEPGTVITKVVAKDRDEG
C. gigas           620 DKGNPPRSSMALVTVHVLDANDNMPRIIYPDNHNNTIKLMYTTPKDSVIARVEADDIDEG
S. purpuratus      609 DDGLSPKTATATVVVTVNDGNDHSPEFIVPSAKNDIRFIPVSADPGLHIMTVESEDEDKD
D. rerio           614 DNGQPSLSATCALYLLVSD-----NLAEVPELKDMSHDESSSKLTFYLIIALVSVSTFFL
M. musculus        641 DHGKPTLSAVAKLIIRSVSGSLPEGVPRVNGEQHHWDMSLPLIVTLSTI-SIILLAAMIT
H. sapiens         614 DHGQPPLSATVTLTVAVAD--RISDILADLGSLEPSAKPNDSDLTLYLVVAAAAVSCVFL

O. vulgaris        681 VVISISICIVRCNNSKNASHRAEIVTPSRIKNEEKYLISRTNNPVINTGNQKEMINKTTH
O. bimaculoides    681 VVISISICIVRCNNSKNASHRAEIVTPSRIKNEEKYLISRTNNPVINTGNQKEMINKTTH
L. gigantea        682 DNARLSYYINKGNVDNT--FHIGSKSGEIVLARRLMLDEREDYHLTVSVQDQGKTQQASQ
C. gigas           680 NNSVLSFYIHKVEPTKPDLFKMNAETGELMIAKTMHLYDSDSYRLVLGVKNGVF---TMF
S. purpuratus      669 ENAAVSYAISHGNTNGAFGIQANGQVVTAQDLEPMWEGVHDITIRATDGGN------PSA
D. rerio           669 TFIIIILAVRFCRRRKPRLLFDGAVAIPSAYLPPNYAEVEGAGTLRSAYNYDAYL---TT
M. musculus        700 IAVKCKRENKEIRTYNCRIAEYSHPQLGGGKGKKKKINKNDIMLVQSEVEERNAMN-VMN
H. sapiens         672 AFVIVLLAHRLRRWHKSRLLQASGGGLASMPGSH-FVGVDGVRAFLQTYSHEVSL--TAD

O. vulgaris        741 SLKSQSHLYPENELENEWRTSTMVKKLPIATQIYSQQVAMTSD-GRRLDENAFFSCDTMS
O. bimaculoides    741 SLKSQSHLYPENELENEWRTSTMVKKLPIATQIYSQQVAMTSD-GRRLDENAFFSCDTMS
L. gigantea        740 TYLTVRIIYV--NVTYIDSTHEDLRYIIIAGVVTGVTVILSIIIVAVILYVRRSDNQRRH
C. gigas           737 ANLNVIITVSNNTVLGAQSPGSGENNIVIVIAIVVVTVILSVAIIAAICIVKYVDRHRQH
S. purpuratus      723 SSTAVLRVVIANEGFKRSLPPANFTALFNLTIDYYLNLGNNAASSRGILNDWPMIVIISL
D. rerio           726 GSRTSDFKFVRSYNDGTLTADLTLKKTALYDLEGLDAEESTSENKQKPPSADWRFTQNQR
M. musculus        759 VVSSPSLATSPMYFDYQTRLPLSSPRSEVMYLKPASN-NLTVPQGHAGCHTSFTGQGTNS
H. sapiens         729 SRKSHLIFPQPNYADTLISQESCEKKGFLSAPQSLLEDKKEPFSQVNFCDECISYLEKNN
```

Table S4: Percentage identity matrix of Ov-PCDH28.

OvPCDH28	Accession number	*Ov*	*Ob*	*Lg*	*Cg*	*Sp*	*Dr*	*Mm*	*Hs*
O. vulgaris	c35066_g15_i1[3]	100	98	26	27	22	25	26	25
O. bimaculoides	Ocbimv22000847	98	100	26	28	22	25	26	26
L. gigantea	LotgiT51526	26	26	100	32	28	25	25	23
C. gigas	EKC42706	27	28	32	100	28	26	26	26
S. purpuratus	SPU_025622	22	22	28	28	100	27	28	27
D. rerio	ENSDART00000111335	25	25	25	26	27	100	34	42
M. musculus	ENSMUSG00000035566	26	26	25	26	28	34	100	35
H. sapiens	ENSG00000204956	25	26	23	26	27	42	35	100

[3] For the corresponding GenBank Accession Number refer to table S8.

Ov-PCDH50-c32730_g4_i1

```
                                 EC2
O. vulgaris        1   SDPFRLDVTTRPDGSSDLYLYLDGKLDRETKQGYKVRILAEDNGKPPKKSSLDVNIVVAD
O. bimaculoides    1   SDPFRLDVTTRPDGSSDLYLYLDGKLDRETKPGYTVRILAEDNGKPPKKSSLDVKIIVAD
L. gigantea        1   SGLFSLKVVENWDGSSDLGIVIKHPLDRETRDRFQVKVIAKDGGYPVRTGSVIIDITVTD
C. gigas           1   NEMFGLKEIKNIDGTTDLGLVVRYKLDRETLDFYQVEIVAKDGGFPQRSGTVMVNITVID
S. purpuratus      1   AETFGLVQNEFPGGLIIIQLEVIGSLDRENKDNYVMTLYADDGGDPVLSGVTTLNVTVLD
D. rerio           1   QSAFGLDIVETPEGEKWPQLIVQQNLDREQKDTYVMKVKVEDGGNPQKSSTAILQVTVTD
M. musculus        1   QSVFGLDIVETPEGEKWPQLIVQQNLDREQKDTYVMKIKVEDGGTPQKSSTAILQVTVSD
H. sapiens         1   QSVFGLDIVETPEGEKWPQLIVQQNLDREQKDTYVMKIKVEDGGTPQKSSTAILQVTVSD

                                                   EC3
O. vulgaris        61  VNDNAPVFEKSKYNVTTENEVNKSRAIVYVKANDADSGKNGQVSYKFSPRTSSGAKKLFE
O. bimaculoides    61  VNDNAPVFEKAKYNVTTENEVNKSKAIVYVKANDADSGKNGQVSYKFSSRTSSVAKKLFE
L. gigantea        61  VNDNRPVFLNTTYNISVYENIPYNRTVLQLVAIDTDAGSNSELTYRFSSRVNNKIKEAFN
C. gigas           61  DNDNKPLFSQAKYDASIPENHPVGKNVLTLSAQDLDINENGEFTFAFNSRVPQKIKDKFA
S. purpuratus      61  SDDHSPVFDRTSYQVSVAENIGVGQHIIQVRASDPDTGTNGQIIYDFGGSVSAKIIELFE
D. rerio           61  VNDNRPVFKESQIEVHIPENSPVGTSVVQLQATDADVGANAEIKYMFGAQVSPATRRLFA
M. musculus        61  VNDNRPVFKEGQVEVHIPENAPVGTSVIQLHATDADIGSNAEIRYIFGAQVAPATKRLFA
H. sapiens         61  VNDNRPVFKEGQVEVHIPENAPVGTSVIQLHATDADIGSNAEIRYIFGAQVAPATKRLFA

O. vulgaris        121 LDANTGAIYLSQKISVEQPTKYRLFVEAVDGAERPLSAQVVVHVQIIHSQNNPPKISINF
O. bimaculoides    121 LDEDTGAIYLSQKISVEQPTKYKLFVEAVDGAERPLSAQVVVHVQIIHSQNNPPKISINF
L. gigantea        121 IDSKTGRIYAVGKINYEETKQYQFMVEAVDSGTPPLSSQALVTIDIKDENDNVPQININL
C. gigas           121 VNKTSGEIYTISEIDYEEEDNYQFLVEVQDKGREPKSSTSVVNIIILDVNDNAPQISVNL
S. purpuratus      121 IDSESGWLSVKSELDFEDESSHQVSIRATNNVPNPLPDFTTVTVNLIDVNDNKPRLTISA
D. rerio           121 LNTTTGLITVQRPLDREETAIHKLTVLASDGSSSPAR--ATITINVTDVNDNAPNIDLRY
M. musculus        121 LNNTTGLITVQRSLDREETAIHKVTVLASDGSSTPAR--ATVTINVTDVNDNPPNIDLRY
H. sapiens         121 LNNTTGLITVQRSLDREETAIHKVTVLASDGSSTPAR--ATVTINVTDVNDNPPNIDLRY

                                           EC4
O. vulgaris        181 VS----STAKITEGANSESFVAYVQVKDPDVGENGKVGCTLMHEY--FRLRSLNEDDYEV
O. bimaculoides    181 VS----HTAKITEGANSESFVAYVQVKDPDIGENGKVGCALTHEY--FRLRSLDKDDYEV
L. gigantea        181 TP----EGTDISEAVDTKKFVAHVSVSDKDDGDNKHVVCTMSDSH--FILENFFDDSYKI
C. gigas           181 LP----DGTDILESAEVGRYVANFAVSDLDSGPNGEIQCQVLGEF--FKIEEIFNNMYKV
S. purpuratus      181 LGDG-GRFKHIAENSPEDVDVAYVRVTDMDTGVNGQAILTLEDDFGHFYLESFREGQYFL
D. rerio           179 IISPTNGTVMLSEKDPINTKIALITVSDKDTDVNGKVICFIEKDVP-FHLKAVYDNQYLL
M. musculus        179 IISPINGTVYLSEKDPVNTKIALITVSDKDTDVNGKVICFIEREVP-FHLKAVYDNQYLL
H. sapiens         179 IISPINGTVYLSEKDPVNTKIALITVSDKDTDVNGKVICFIEREVP-FHLKAVYDNQYLL

O. vulgaris        235 VIKKPVDRETNEHFNVTITCRDQGSPPLQSESNFRVEVDDINDEYPVFERSIYDVTFEEN
O. bimaculoides    235 VIKKPVDRETNEHFNVTITCRDQGSPPLQSESNFRVEVDDINDEYPQFERPVYDVTFEEN
L. gigantea        235 VLGKKLDYETQTSHNVTVTCRDNGRVPLENSSSFIVHVLDENDNFPEFSQTVYKGSIIEN
C. gigas           235 IIKSPLDYESRHVHNVTIQCQDQGIPQHQNTSSFLINVLDVNDNNPVFLQSIYRATIKEN
S. purpuratus      240 KTAGVLDREDIDFYNITILAEDRGSPVLSSRRRFAVFVDDENDNSPIFSSSVYHATISEN
D. rerio           238 ETSALLDYEGTKEYIFKIVASDSGKPSLNQTALVRVRLEDENDNPPIFTQPVIELAVMEN
M. musculus        238 ETSSLLDYEGTKEFSFKIVASDSGKPSLNQTALVRVKLEDENDNPPIFNQPVIELSVSEN
H. sapiens         238 ETSSLLDYEGTKEFSFKIVASDSGKPSLNQTALVRVKLEDENDNPPIFNQPVIELSVSEN

                                EC5
O. vulgaris        295 SIIGIKVEAVSARDKDIGQNGEVRYFLDREALPYFIVDPQTGIIRTVTVFDRESTSKKEF
O. bimaculoides    295 SIIGIKVEAVSARDKDIGQNGEVRYFFDRDALPYFIVDPQTGIIRTVTVFDRESTSKKEF
L. gigantea        295 NAISEEILQVSARDKDNGENGRVGYSLDNQASQFFFIDRDSGIITAKVRLDREDIPEFKF
C. gigas           295 NPPNEVITTVKAVDKDSGLAGKVTYFMHTDGSDSFHVDSTSGVVTVKKSLDREISPVILF
S. purpuratus      300 NEPGHRVATVQAIDKDELENGEVVYSLLDDKDGSFGIHPFNGVLTANVSLDREDGESIDL
D. rerio           298 NLRDMFLTTISATDEDSGRNAEIVYQL-GPNASFFDLDRKTGVLTASRVFDREEQERFLF
M. musculus        298 NRRGLYLTTISATDEDSGKNADIVYQL-GPNASFFDLDRKTGVLTASRVFNREEQERFIF
H. sapiens         298 NRRGLYLTTISATDEDSGKNADIVYQL-GPNASFFDLDRKTGVLTASRVFDREEQERFIF

                                                                           EC6
O. vulgaris        355 KIYAKDLGEPSHTSSATMRVNVLDVNDEAPVFTQDLFHFKTYENQLPKFPVGFINASDRD
O. bimaculoides    355 KIYAKDLGKPSHTSSATMRVNVLDVNDEAPVFTEKLFHFKTYENQLPKFPVGFINATDRD
L. gigantea        355 NVIATDYGKPPKSQSVNVIVTVLDQNDQPPKFQRPVFFCYVMENLNPGASAGNVTAIDKD
C. gigas           355 HVNASDAGNPQLKSSTLIRLTLEDENDNTPMFKKSHFEFYVLEEQKNLPIVGRLFAEDPD
S. purpuratus      360 MIRACDRGQPQGCSDVPLTVRVLDMNDNGPTFGGDLIEMRIDENKPSGTIVGRAVATDAD
D. rerio           357 TVTARDNGTRALQSQAAVIVTILDENDNSPKFTHNHFQFFVSENLPKYSTVGVITVTDAD
M. musculus        357 TVTARDNGTPPLQSQAAVIVTVLDENDNSPKFTHNHFQFFVSENLPKYSTVGVITVTDED
H. sapiens         357 TVTARDNGTPPLQSQAAVIVTVLDENDNSPKFTHNHFQFFVSENLPKYSTVGVITVTDAD
```

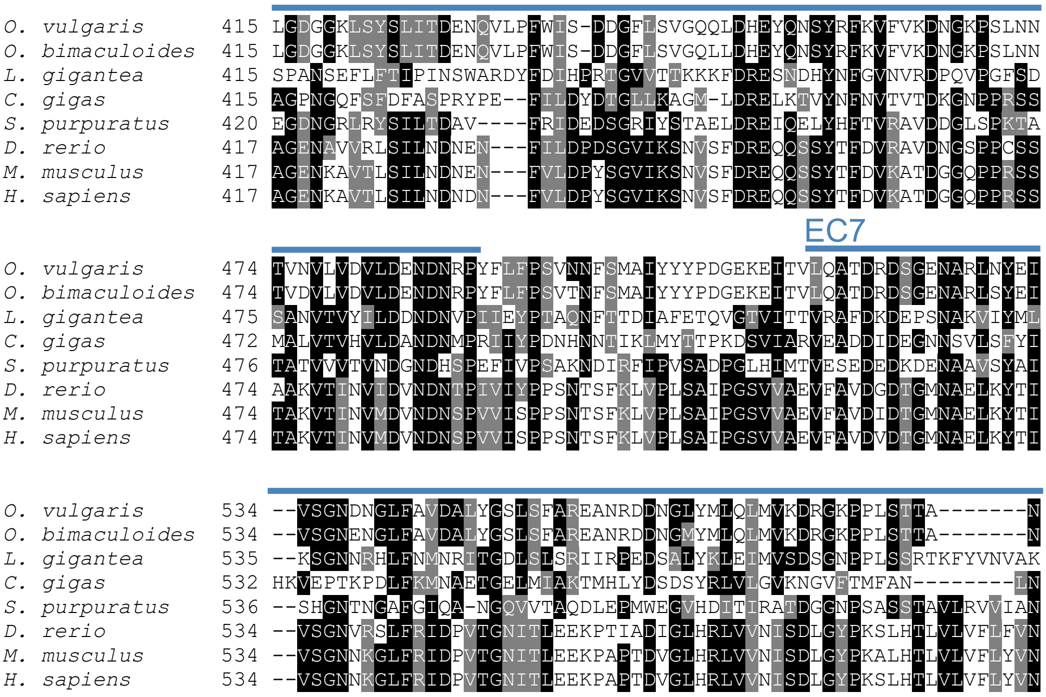

Table S5: Percentage identity matrix of Ov-PCDH50.

Ov-Pcdh50	Accession number	*Ov*	*Ob*	*Lg*	*Cg*	*Sp*	*Dr*	*Mm*	*Hs*
O. vulgaris	c32730_g4_i1[4]	100	95	36	34	33	30	30	30
O. bimaculoides	Ocbimv22013746	95	100	36	34	33	30	30	31
L. gigantea	LotgiT62221	36	36	100	39	32	33	33	33
C. gigas	EKC42706	34	34	39	100	31	32	33	33
S. purpuratus	SPU_025622	33	33	32	31	100	36	36	36
D. rerio	ENSDARG00000111493	30	30	33	32	36	100	88	88
M. musculus	ENSMUSG00000055421	30	30	33	33	36	88	100	99
H. sapiens	ENSG00000184226	30	31	33	33	36	88	99	100

[4] For the corresponding GenBank Accession Number refer to table S8

Ov-PCDH52-c31207_g1_i5

```
                                                 EC1
O. vulgaris        1  L--NFHVLEETAEGTYVGNVAKAF-------TRPTHGDYLFKFLT-QGNQYTNLFHIGTN
O. bimaculoides    1  L--NFHVLEETAEGTYVGNVAKAF-------TRPTHGDYLFKFLT-QGNQYTNLFHIGTN
L. gigantea        1  MEASFHLVESEPPETIVGNIASKINIAR-GLSKSEFNSLRYSFLNPNDDSIASLFNINPE
C. gigas           1  FVADFSINEQGPPGQLIGNIATKSNFL----QKANHSSVTYTYLDKT-NAYAGLFSITES
S. purpuratus      1  RDIFYDIDEGVGPGTVIGNVADDLAI-------TIDANTEFSMLGVPNE--TAYVSLDSQ
D. rerio           1  L--HFSVPEEQERGTVVGNIAEDLGL--------DITKISARRFQTVPSSRTPYLEVNLE
M. musculus        1  L--IYTIREELPENVPIGNIPKDLNISHINAATGTSASLVYRLVSKAGD--APLVKVSSS
H. sapiens         1  L--IYTIREELPENVPIGNIPKDLNISHINAATGTSASLVYRLVSKAGD--APLVKVSSS

O. vulgaris       51  SGII-TTAVPIDREHICDNDMGGHTCIVTVSVAVQSSSDPRFLDMMNIKINIDDINDNAP
O. bimaculoides   51  SGII-TTAVPIDREHICDNDIGGHTCIVTVSVAVQSSSDPRFLDMMNIKINIDDINDNAP
L. gigantea       60  NSDV-STVEKIDREKVCEFTS---ECVITFDIKISSLVTSFF-EIVTVKIIIDDLNDNPP
C. gigas          56  NGDL-TTTTTIDRENIQCKDP---QCILTFDVGANFGDF----DVITVNVHVIDINDNAP
S. purpuratus     52  TGEL-TTVLDLDREELCPGSS----ALCEIEVNAIELGTR---EVITVKVTINDINDHAP
D. rerio          51  NGAL-VVNERIDREEICRQTV---PCLLHLEVFLENPL-----ELFRVEIEVMDINDNPP
M. musculus       57  TGEIFTTSNRIDREKLCAGASYAEENECFFELEVVILPNDFF-RLIKIKIIVKDTNDNAP
H. sapiens        57  TGEIFTTSNRIDREKLCAGASYAEENECFFELEVVILPNDFF-RLIKIKIIVKDTNDNAP
                                            EC2
O. vulgaris      110  LFDKDEIALEISESTPANTKFPIEDAYDLDTGIDNSIKYYILLNDRDKFTLLQENGFLDG
O. bimaculoides  110  LFDKDEIALEISESTPANTKFPIEDAYDLDTGIDNSIKYYILLNDRDKFTLLQENGFLDG
L. gigantea      115  KFPELEITVFIPENVNPGSTYRINGATDLDRGQNNSVQLYEMVSSAN-LFDLKVDKKLDG
C. gigas         108  QFPKSLITINISETSSVGHLVQLPSAVDLDTGENNGVQNYEIFPANV-TFGLQTKKKLDG
S. purpuratus    104  EFRDDLTNMSIPESVVPGTRFPLSTASDEDIGE-NAIQGYRLSDEYAETFGLVQNEFPGG
D. rerio         102  SFPETDITVEITESATPGTRFPVENAFDPDVGT-NALSTYAITTNN--YFYLDVQTQGDG
M. musculus      116  MFPSPVINISIPENTLINSRFPIPSATDPDTGF-NGVQHYELLNGQS-VFGLDIVETPEG
H. sapiens       116  MFPSPVINISIPENTLINSRFPIPSATDPDTGF-NGVQHYELLNGQS-VFGLDIVETPEG

O. vulgaris      170  -----LQLKQKLDHEEQDFYQLVIVAKDNGTPQRSGNVVVNITVLDANDNAPKFDKKSYT
O. bimaculoides  170  -----LQLKQKLDHEEQDFYQLVIVAKDNGTPQRSGNVVVNITVLDANDNAPKFDKKSYT
L. gigantea      174  TSDLRIVVKNVIDREKKHYYRFFIVAKDGGRPPLSGNVTVNVNITDENDNSPEFTEEIYD
C. gigas         167  SFDVKLVLLENLDREKKAFYTCKIFAKDGGVEQNIGTLQVDINVLDDNDNPPVFGESIYN
S. purpuratus    163  LIIIQLEVIGSLDRENKDNYVMTLYADDGGDPVLSGVTTLNVTVLDSDDHSPVFDRTSYQ
D. rerio         159  NRFAELVLDKPLDREQQAIHKYVLTAVDGGQPQRTGTALLVVKVLDSNDNAPTFDQSVYS
M. musculus      174  EKWPQLIVQQNLDREQKDTYVMKIKVEDGGTPQKSSTAILQVTVSDVNDNRPVFKEGQVE
H. sapiens       174  EKWPQLIVQQNLDREQKDTYVMKIKVEDGGTPQKSSTAILQVTVSDVNDNRPVFKEGQVE
                                   EC3
O. vulgaris      225  VYIHENRSILSTILTLHAEDLDSGPNGQVGYKLHHRTSSKIKEIFDVNQTTGEIHLISRV
O. bimaculoides  225  VYIHENRSILSTILTLHAEDLDSGPNGQVGYKLHHRTSSKIKEIFDVNQTTGEIHLISRV
L. gigantea      234  VSVTENTPIYSVIAKIHAIDRDSDSNGKVSYRFSTLKNPDIEKLFALNPLSGDITVKNEL
C. gigas         227  KTVPENTLPGTTILRVTAIDADSGLNGELEYHIS---QGAYSDIFSINNRTGEILLLKKL
S. purpuratus    223  VSVAENIGVGQHIIQVRASDPDTGTNGQIIYDFGGSVSAKIIELFEIDSESGWLSVKSEL
D. rerio         219  VSIRENSPVGTLVIQLNASDMDEGQNGEIVYSLSSHNSPRIRDLFNIDSRTGRIEVTGEV
M. musculus      234  VHIPENAPVGTSVIQLHAIDADIGSNAEIRYIFGAQVAPATKRLFALNNTTGLITVQRSL
H. sapiens       234  VHIPENAPVGTSVIQLHAIDADIGSNAEIRYIFGAQVAPATKRLFALNNTTGLITVQRSL

O. vulgaris      285  DYEDRPRYSFNVIAYDHGAIVQSSTASVNVYVVDVNDNKPEIIINLLS--LGYAANVSES
O. bimaculoides  285  DYEDRPRYSFNVIAYDHGAIVQSSTASVNVYVVDVNDNKPEIIINLLS--LGYAANVSES
L. gigantea      294  QYQSGKQFETIVEAFDQGTPPQVGQAKLILRIIDVGNNPPIITVNPVSDVVGDMILLPEG
C. gigas         284  VYEPNEIFSFFVEARDKGAIPNYAQVKVNIQIQDAGNNPPVVKVNLVSGSAG-KVLISEL
S. purpuratus    283  DFEDESSHQVSIRATNNVPNPLPDFTTVTVNLIDVNDNKPRLTISALGDG-GRFKHIAEN
D. rerio         279  DYEESSTHQIYVQAKDMGPNAVPAHCKVLVKLIDVNDNTPEISFSTV------TESVSEQ
M. musculus      294  DREETAIHKVTVLASDG--SSTPARATVTINVTDVNDNPPNIDLRYIISPINGTVYLSEK
H. sapiens       294  DREETAIHKVTVLASDG--SSTPARATVTINVTDVNDNPPNIDLRYIISPINGTVYLSEK
                             EC4
O. vulgaris      343  ASKGKFIAHVSINDRDQDQNGNVSCSVNDH--HFSLQIFSIKTYKVVVAKPLDFEKTSVH
O. bimaculoides  343  ASKGKFIAHVSINDRDQDQNGNVSCSVNDH--HFSLQIFSIKTYKVVVAKPLDFEKTSVH
L. gigantea      354  ARIGTVVAHVNIDDKDQGPNGDVLCSCLHE--YFSVHKIEGRGYIVQVKKPLDRELVDEL
C. gigas         343  INIDAFVAHVSVEDSDTGKNGEYVCSISSS--FFDIKPLQSKGYKVVVKIPLDREKASEH
S. purpuratus    342  SPEDVDVAYVRVTDMDTGVNGQAILTLEDDFGHFYLESFREGQYFLKTAGVLDREDIDFY
D. rerio         333  AAPGTVIALLSVTDRDSGENGQMTCELHGEV-PFKLKSSFKNYYTIVTDGPLDREKAESY
M. musculus      352  DPVNTKIALITVSDKDTDVNGKVICFIEREV-PFHLKAVYDNQYLLETSSLLDYEGTKEF
H. sapiens       352  DPVNTKIALITVSDKDTDVNGKVICFIEREV-PFHLKAVYDNQYLLETSSLLDYEGTKEF
```

EC5

```
O. vulgaris        401 NVSIRCHDHGTPQLHSEKSFLVIVADKNDNPPVFEQTLIKTPITENNNFHDYVTKVTAKD
O. bimaculoides    401 NVSIRCHDHGTPQLHSEKSFLVIVADKNDNPPVFEQTLIKTPITENNNFHDYVTKVTAKD
L. gigantea        412 KVTVVCRDSGTPRLSARTKFRIKLTDENDNPPIFTKRVYETTLEENNVIDRPLIRVSAVD
C. gigas           401 NVTVSCHDLGTPPLYSSVSFIVQVGDENDNKPVFLQPTYFASIEENNPVGVTVTKVSATD
S. purpuratus      402 NITILAEDRGSPVLSSRRRFAVFVDDENDNSPIFSSSVYHATISENNEPGHRVATVQAID
D. rerio           392 TLTVVAKDKGVPSLSTSKSIKVHVSDENDNAPRFMQSVYDVYVTENNVPGAYIYAVSAVD
M. musculus        411 SFKIVASDSGKPSLNQTALVRVKLEDENDNPPIFNQPVIELSVSENNRRGLYLTTISATD
H. sapiens         411 SFKIVASDSGKPSLNQTALVRVKLEDENDNPPIFNQPVIELSVSENNRRGLYLTTISATD

O. vulgaris        461 NDTGINSEIHYELHH-DAHS-----WFNIDHRTGVITANKQFDREQNAEIKFRVLAIDSG
O. bimaculoides    461 NDTGINSEIHYELHH-DAHS-----WFNIDHRTGVITANKQFDREQNAEIKFRVLAIDSG
L. gigantea        472 DDLGRNAIVHYRLKL-DDQG-----MFKINTNTGEIVANDVFNRETISEMSFTVLAIDEG
C. gigas           461 NDIDLNAQIQYVLHSD-AGS-----SFSINRITGVINATGVLDRETTSDYLFRVLAVDGG
S. purpuratus      462 KDELENGEVVYSLLD-DKDG-----SFGIHPFNGVLTANVSLDREDGESIDLMIRACDRG
D. rerio           452 PDVGQNAYVTYSILECEIQGMSILTYVSINSENGYLYALRSFDYEQIKEFSFMVHAKDSG
M. musculus        471 EDSGKNADIVYQLG--PNAS-----FFDLDRKTGVLTASRVFNREEQERFIFTVTARDNG
H. sapiens         471 EDSGKNADIVYQLG--PNAS-----FFDLDRKTGVLTASRVFDREEQERFIFTVTARDNG
```

EC6

```
O. vulgaris        515 SPPLTGTATVHLNILDINDEAPQF-----KSGYYKFHVKEDQEPFTYIGRVDAQDLDHGE
O. bimaculoides    515 SPPLTGTATVHLNILDINDEAPQF-----KRGYYKFHVKEDQEPFTFIGRVDAQDLDHGE
L. gigantea        526 TPPMTGTAEVLVRIIDQNDNSPKF-----SPAALIFSVREDTRAGGEVGMLHATDADFNF
C. gigas           515 KEPKTGTATVIVNITDINDNKPKF-----SKSLFTFEVSELSDPYTHVGRVTAQDPDLGQ
S. purpuratus      516 QPQGCSDVPLTVRVLDMNDNGPTF-----GGDLIEMRIDENKPSGTIVGRAVATDADEGD
D. rerio           512 APELTANATVNVIIVDQNDNAPSVIAPLGKNGTAREHLPRSAEPGYLVTRIVATDADDGE
M. musculus        524 TPPLQSQAAVIVTVLDENDNSPKF-----THNHFQFFVSENLPKYSTVGVITVTDEDAGE
H. sapiens         524 TPPLQSQAAVIVTVLDENDNSPKF-----THNHFQFFVSENLPKYSTVGVITVTDADAGE

O. vulgaris        570 NGRIRYWISKNYEAPQLFM-VTDGVLKTKARLS--RMKDQRYDFLVVAADHGKKPLSSSA
O. bimaculoides    570 NGRIRYWISKNYEAPQLFM-VTDGVLKTKARLS--RMKGQRYDFLVVAADHGKKPLSSST
L. gigantea        581 NADIEYTMIVSGDSLPFVV-FSNGVIRTDRELD--FESQKRFTFKIIAKDKGSPPRNTTA
C. gigas           570 NAYLQYSMSSQDRNVPFEV-SLNGTVITRAELD--REYMGQYNFMVYVRDSGNPSLNSSA
S. purpuratus      571 NGRLRYSILTD---AVFRIDEDSGRIYSTAELD--REIQELYHFTVRAVDDGLSPKTATA
D. rerio           572 NARLSYSILRGNELGMFRMDWRTGELRTARRVSSKRDPPHPYDLLIEVRDHGQPPLSSSA
M. musculus        579 NKAVTLSILNDNENFVLDP--YSGVIKSNVSFD--REQQSSYTFDVKATDGGQPPRSSTA
H. sapiens         579 NKAVTLSILNDNDNFVLDP--YSGVIKSNVSFD--REQQSSYTFDVKATDGGQPPRSSTA
```

EC7

```
O. vulgaris        627 SVTVIVDD------VNDNYPYIIYPSDTNNTISVIYDMTPVD-RPISRVVALDDDEGENA
O. bimaculoides    627 NVTVIVDD------VNDNYPYIIYPSDTNNTISVIYDMTPVD-RPISRVIALDDDEGENA
L. gigantea        638 TVTVYVSD------SNDHSPVIVFPTDNNNTVTIRSDIEPGT-V-ISRVVASDSDAGENA
C. gigas           627 YVTVYVMD------ANDNAPRILFPNKMNNTVSSTVTSLP-----ITKIIAEDKDDGINR
S. purpuratus      626 TVVVTVND------GNDHSPEFIVPSAKNDIRFIPVSADPGL-H-IMTVESEDEDKDENA
D. rerio           632 SINVLLVDSVVEGRSGDRGSVKSKEGSLDLTLILIIALGSVSFIFLLAMIVLAVRCQKDK
M. musculus        635 KVTINVMD------VNDNSPVVISPPSNTSFKLVPLSAIPGS--VVAEVFAVDIDTGMNA
H. sapiens         635 KVTINVMD------VNDNSPVVISPPSNTSFKLVPLSAIPGS--VVAEVFAVDVDTGMNA

O. vulgaris        680 MLSYFLKSTPETAGLFRINHTSGEIMLIKDSDIRIFSKSY---KLPIVVKDHGKPPKSTS
O. bimaculoides    680 MLSYFLKSTPETAGLFRINHTSGEIMLIKDSDIRIFSKSY---KLPIVVKDHGKPPKSTS
L. gigantea        690 VLSFYINSGNEQ-NMFHIGTKTGEIVLAKRIRIYESTEYY----LSISVQDQGTTQQASE
C. gigas           676 QLTYFIQTGNDK-NMFNLHSQTGELFLNHNYQFTTDQSV----SLIICVQDGGLPVKSAC
S. purpuratus      678 AVSYAISHGNTN-GAFGI-QANGQVVTAQDLEP-MWEGVH---DITIRATDGGNPSASST
D. rerio           692 KLNIYTCMAGDSCCQCCSRQARGRKKKLSKSDIMLVQSTNVASTAQVPVEESGSFGSHHQ
M. musculus        687 ELKYTIVSGNNK-GLFRIDPVTGNITLEEKPAP-TDVGLH---RLVVNISDLGYPKALHT
H. sapiens         687 ELKYTIVSGNNK-GLFRIDPVTGNITLEEKPAP-TDVGLH---RLVVNISDLGYPKSLHT
```

```
                                                                                TM
O. vulgaris      737 AILIVMMI----------------LSNSTELPYGNSTPDGERNLTIVIVLAAVTVVLATC
O. bimaculoides  737 AILIVMMI----------------LSNSTELPYGNGTPDGERNLTIVIVLAAVTVVLATC
L. gigantea      745 AHLTVLV------------------DFVNSTALEMARERDLQYIIIAGVVTGVTIILSII
C. gigas         731 TQLNVEV------------------SINNATIADEKPETDNKYIYISIGVIIFTLIISAG
S. purpuratus    732 AVLRVVIANEGFKRSLPPANFTALFNLTIDYYLNLGNNAASSRGILNDWPMIVIISLAGC
D. rerio         752 NQNYCYQV-CLTPE-----------SAKTDLMFLKPCSPSRSTDTEHNPCGAIVTGYADQ
M. musculus      742 LVLVFLYVNDTAGNT---SYIYDLIRRTMETPLDRNIGDSGQPYQNEDYLTIMIAIVAGA
H. sapiens       742 LVLVFLYVNDTAGNA---SYIYDLIRRTMETPLDRNIGDSSQPYQNEDYLTIMIAIIAGA

O. vulgaris      781 IVITICIIKKVDQNRRR---YPAKM---HEDARRKSDSSPDS-----DRQKHKKEVSFSL
O. bimaculoides  781 IVITICIIKKVDQNRRR---YPAKM---HEDARRKSDSSPDS-----DRQKHKKEVSFSL
L. gigantea      787 IVAVIIYVRRSDLQRRR--GQTICV---QEHGENKNVEKQLWHSVPKDEMVAPSDKTTLD
C. gigas         773 IVVVILVIRKKDSERKNNKSSPGPLGVPMQNGYAPTGTSTLSNSHPEAEYMERKKVTLNA
S. purpuratus    792 AVILVIVFLLVAARCRTKNREQGK----YIVPSGEELFATRQAAKPADNSGKTSDTDSGL
D. rerio         800 QPDIISNGSILSSETKHQRTELSYL---VDRPRRVNSSAFQEADIVSSKDSGHGDSEQGD
M. musculus      799 MVVIVVIFVTVLVRCRHASRFKAA----QRSKQGAEWMSPNQ-----ENKQNKKKKRKKR
H. sapiens       799 MVVIVVIFVTVLVRCRHASRFKAA----QRSKQGAEWMSPNQ-----ENKQNKKKKRKKR

O. vulgaris      830 DGADPTSKGSANSLASNTTVKNPGAFFAPDLPSLQ-------------------------
O. bimaculoides  830 DGADPTSKGSANSLASNTTVKNPGAFFAPDLPSLQKCEDAGWKAERFPFEVGCRGFVGHS
L. gigantea      842 EFFKEKNCEKPDGDDSYFKNKSMDHFSGQQFFTFRKVSHFEFYSSYVLYCSTCVFISGRL
C. gigas         833 GTNREGSLGSSSHNYS-SNISQNVSTNGRAHPCDSNSDTSGETIPSDSGRGGSEDDNPST
S. purpuratus    848 TSTASSSSNIPSKNIRKWRAQQDRDRDSMGSSNPGSTTNLGTMPPGITGVANVDLRLGNR
D. rerio         857 SDHDATNRGHTAGADLFSNCTEECKALGHSDRCWMPSFMPGDSRQGADYRSNLHVPGMDA
M. musculus      850 KSPKSSLLNFVT-------IEESKPDDAVHEPINGTISLPAELEEQGIGRFD----WGPA
H. sapiens       850 KSPKSSLLNFVT-------IEESKPDDAVHEPINGTISLPAELEEQSIGRFD----WGPA
```

Table S6: Percentage identity matrix of Ov-PCDH52*.*

Ov-Pcdh52	Accession number	*Ov*	*Ob*	*Lg*	*Cg*	*Sp*	*Dr*	*Mm*	*Hs*
O. vulgaris	c31207_g1_i5[5]	100	99	32	35	27	27	29	29
O. bimaculoides	Ocbimv22020908	99	100	31	34	26	26	28	28
L. gigantea	LotgiT105112	32	31	100	35	26	24	28	28
C. gigas	EKC18334	35	34	35	100	26	25	28	28
S. purpuratus	SPU_025622	27	26	26	26	100	25	32	32
D. rerio	ENSDARG00000099729	27	26	24	25	25	100	25	25
M. musculus	ENSMUSG00000055421	29	28	28	28	32	25	100	99
H. sapiens	ENSG00000184226	29	28	28	28	32	25	99	100

[5] For the corresponding GenBank Accession Number refer to table S8

Ov-DSCAM-c34599_g6_i1

```
                                                                       Ig1
O. vulgaris         1 MLNVSKFGLTGLFILR-QMYILVAALTTEEPPADTNGPVFTEDVPSTVVFANTKGASIDC
O. bimaculoides     1 ------------------------------------------------------------
L. gigantea         1 ------------------------------------------------------------
C. gigas            1 ---------------M-ELIAGRRAKTTESPFDIIGGPIFLTEPPSSLDFANTKGASVQC
D. melanogaster     1 ------MDLHSLFKAILILYVIRAETSQFVNGLDLQGPIFLHEPPHRVEFSNNSGGLIEC
S. purpuratus       1 ------------------------------------------------------------
D. rerio            1 -----------MWILAIIFFQCILNVLSED---LHSSLYFVNASLQEVVFASTTGTLVPC
M. musculus         1 -----------MWILALSLFQSFANVFSEE---PHSSLYFVNASLQEVVFASTSGTLVPC
H. sapiens          1 -----------MWILALSLFQSFANVFSED---LHSSLYFVNASLQEVVFASTTGTLVPC

O. vulgaris        60 TAHGLPPPKLNWIRKDGTVVKDIPKVLQVLPNNTLYFLPFEPNLFQPDAHSQEYRCIAKN
O. bimaculoides     1 ------------------------------------------------------------
L. gigantea         1 ------------------------------------------------------------
C. gigas           45 TAHGQPAPTLDWVKDDDTPVEDVSQILKVLPNNTLHFYPFKRSDFQSKVHAASYRCIASN
D. melanogaster    55 SGHGSPPPEVEWTPI-----PPQQDMVFQLSNGSMMFYPFTAEKYRHEVHATVYRCKLRN
S. purpuratus       1 ------------------------------------------------------------
D. rerio           47 PAAAVPPATLRWYLATGEESYNVPGIRHVHPNGTLQIFHFPPSSFSKVIHDNTYYCTAEN
M. musculus        47 PAAGIPPVTLRWYLATGEEIYDVPGIRHVHPNGTLQIFPFPPSSFSTLIHDNTYYCTAEN
H. sapiens         47 PAAGIPPVTLRWYLATGEEIYDVPGIRHVHPNGTLQIFPFPPSSFSTLIHDNTYYCTAEN
                                      Ig2
O. vulgaris       120 KGGKIISRPLRITAVVVDRYKVYNVQLNDVWVISNGTAILKCLINPRFVNEYIKVTKWTQ
O. bimaculoides     1 ------------------------------------------------------------
L. gigantea         1 ------------------------------------------------------------
C. gigas          105 SGGRISSRSMRVKAVRVEDYVTYDLQLSDVWSVRGSTAVFKCIMNPYYVKDYIHVVGWSK
D. melanogaster   110 LVGTVLSREVHVRGVVNQK---YAVQVHDEYVMTGNTAVLKCQV-PSYMSEFVLVTAWVQ
S. purpuratus       1 ------------------------------------------------------------
D. rerio          107 PSGKIRSQDVHIKAVLREP---YTVRVADQTAMRGSVAVFKCII-PSSVENYITVVSWER
M. musculus       107 PSGKIRSQDVHIKAVLREP---YTVRVEDQKTMRGNVAVFKCII-PSSVEAYVTVVSWEK
H. sapiens        107 PSGKIRSQDVHIKAVLREP---YTVRVEDQKTMRGNVAVFKCII-PSSVEAYITVVSWEK

O. vulgaris       180 DSKP-----ITDHDRVSAMNNGELHVRNVQDSDASLVYRCVTRNILTQDTKTSPAASIHV
O. bimaculoides     1 ------------------------------------------------------------
L. gigantea         1 ------------------------------------------------------------
C. gigas          165 GTKP-----IQAGDRISVLSDGELHIRDIRDEDKYSMYTCVARNILTGDEKPSKAAYLHV
D. melanogaster   166 DTGMHLYPNTDIGGKYTVLSNGELYINNAGPNDAYKSYTCRTVNRLTGEVQISTYPGRII
S. purpuratus       1 ------------------------------------------------------------
D. rerio          163 DTVP-----LVSGARFLITSTGALYILDVQKEDELFNYRCMTRHRYTSETRQSNSARLFV
M. musculus       163 DTVS-----LVSGSRFLITSTGALYIKDVQNEDGLYNYRCITRHRYTGETRQSNSARLFV
H. sapiens        163 DTVS-----LVSGSRFLITSTGALYIKDVQNEDGLYNYRCITRHRYTGETRQSNSARLFV
                           Ig3
O. vulgaris       235 HDPPVIWKTPNIETTDT-GLIIQVGKTAELPCVATGNPLPNYKWTKGSKTIVS----DKK
O. bimaculoides     1 ------------------------------------------------------------
L. gigantea         1 ------------------------------------------------------------
C. gigas          220 HDPPASWTPPKIDDIMT-QLTVNEGEKVELPCVASSNPLPKYRWSFSNKELVI----DSV
D. melanogaster   226 VTEPKGMVQPRINVEKHSMRHVVLNGQTTLPCIAQGHPVPTYRWFKEENEQLLPLQLSER
S. purpuratus       1 ------------------------------------------------------------
D. rerio          218 PDPSNS--APAILDGFE-KREVMASHRVELPCKASGHPAPKYRWLKNNRPLES----DSR
M. musculus       218 SDPANS--APSILDGFD-HRKAMAGQRVELPCKALGHPEPDYRWLKDNMPLEL----SGR
H. sapiens        218 SDPANS--APSILDGFD-HRKAMAGQRVELPCKALGHPEPDYRWLKDNMPLEL----SGR
                                                                   Ig4
O. vulgaris       290 KFEQKGGNLVIHNLTVTDSGVYKCGVFNKKGSTSVSIQLTVTSPLSATLYPADQIVDAGR
O. bimaculoides     1 ------------------------------------------------------------
L. gigantea         1 ------------------------------------------------------------
C. gigas          275 SRIQRAGNLVIVDAKVTDSGVYMCNASNSHGSATGTTQLTVQYPLSATIDPQQQKVDSNQ
D. melanogaster   286 ITIVSAGLLKITKARLEDSGKYLCWVNNTAGEETIQVSLTVTAPLTAHLQPQVQTVDVDK
S. purpuratus       1 ------------------------------------------------------------
D. rerio          271 -FRQSVTGLLIERAQPSDTGSYVCEVWNSYGNAEVIGRLTVKEPLKAVVSPRKVKGSVGS
M. musculus       271 -FQKTVTGLLIENSRPSDSGSYVCEVSNRYGTAKVIGRLYVKQPLKATISPRKVKSSVGS
H. sapiens        271 -FQKTVTGLLIENIRPSDSGSYVCEVSNRYGTAKVIGRLYVKQPLKATISPRKVKSSVGS
```

```
O. vulgaris       350 VATFNCSVQGSPINEITWYHNGEPLTNSSRKHII--VDNRLQLPAVNRNDQGMYQCIVGN
O. bimaculoides     1 ------------------------------------------------------------
L. gigantea         1 ------------------------------------------------------------
C. gigas          335 SAIFNCTVLGHPIKSVFWMKDGFKISPNQKVRIT--DQSVLTIHAVNKKDQGMYQCVVKN
D. melanogaster   346 DAQFQCIVSGHPVHDVNWLHDGKPILRDNRVEILT-DPPRLIIKKVQKEDPGMYQCFVSN
S. purpuratus       1 ------------------------------------------------------------
D. rerio          330 QVSLSCSVTGSDEFELSWYRNGDKINTGANIRMNGINKENLVMDGMAKSDGGVYQCFSRK
M. musculus       330 QVSLSCSVTGNEDQELSWYRNGEILNPGKNVRITGLNHANLIMDHMVKSDGGAYQCFVRK
H. sapiens        330 QVSLSCSVTGTEDQELSWYRNGEILNPGKNVRITGINHENLIMDHMVKSDGGAYQCFVRK
                                           Ig5
O. vulgaris       408 DVDMSQATGELRLGAAHPAFLETFDTVLVQPGSNVTFKCAVSGNPPPTIRWTLDDELIYS
O. bimaculoides     1 ------------------------------------------------------------
L. gigantea         1 ------------------------------------------------------------
C. gigas          393 EETSSQGTAQLLLGAAHPTFVDTFINQYAQTGQKTSIRCIATGNPTPDIRWKLDGEILTE
D. melanogaster   405 EWEQIQSTAELQLGDASPELLYWFSEQTLQPGPTVSLKCVATGNPLPQFTWSLDGFPIPD
S. purpuratus       1 ------------------------------------------------------------
D. rerio          390 AKMSAQDFVQVILEDGTPKILSAFSEKVVGPNDFVSLTCHVKGTPQPAITWTLDDEVVAK
M. musculus       390 DKLSAQDYVQVVLEDGTPKIISAFSEKVVSPAEPVSLVCNVKGTPLPTVTWTLDDDPILK
H. sapiens        390 DKLSAQDYVQVVLEDGTPKIISAFSEKVVSPAEPVSLMCNVKGTPLPTITWTLDDDPILK
                                                                           Ig6
O. vulgaris       468 SDITTIKTYATEQADVVSTLHITNIQVQTGGEYRCIGSNVVGEVTHMARANVYGVPYVRV
O. bimaculoides     1 ------------------------------------------------------------
L. gigantea         1 ------------------------------------------------------------
C. gigas          453 NSDIKIGSFLNPAGDVVSYVNISNVHLHYGGEYQCISSNEVGEIHHVGQMFVYGVPFVRP
D. melanogaster   465 SSRFLVGQYVTIHDDVISHVNISNVKEEDGGEYTCTAQNAIGKVSHSAKVNIYGLPYIRE
S. purpuratus       1 ------------------------------------------------------------
D. rerio          450 DSRHRIVHSITAEGNVVSYLNISHIQVRDSGVYRCTCNNSAGTVSYQARINVRGSADIRP
M. musculus       450 GSGHRISQMITSEGNVVSYLNISSSQVRDGGVYRCTANNSAGVVLYQARINVRGPASIRP
H. sapiens        450 GGSHRISQMITSEGNVVSYLNISSSQVRDGGVYRCTANNSAGVVLYQARINVRGPASIRP

O. vulgaris       528 LQNITATANQPLIMKCYVSGYPIGSITWFKASQALPIDHRQKV-V-NGTLTVEDVQSAYD
O. bimaculoides     1 ------------------------------------------------------------
L. gigantea         1 ---------MKLSIRCYVSGYPVNTIKWYRDGHMLPMNHLQKV-E-DGTLIIEKVQRNYD
C. gigas          513 MENVTTTAGKNFSIRCYVAGYPVSQVTWSKGHNVLPKNHLQKV-V-NDTLFIEGVQKDHD
D. melanogaster   525 MPKITGISGSDLIVKCPVAGYPIDKIHWERDGQTLPINRRQRAYN-NGTLIIEQLQRLED
S. purpuratus       1 ------------------------------------------------------------
D. rerio          510 MKNLTAIAGWDMYIHCHVIGYPYYSIKWFKNSNLLPFNDRQRAFENNGTLKLLNVQKELD
M. musculus       510 MKNITAIAGRDTYIHCRVIGYPYYSIKWYKNANLLPFNHRQVAFENNGTLKLSDVQKEVD
H. sapiens        510 MKNITAIAGRDTYIHCRVIGYPYYSIKWYKNSNLLPFNHRQVAFENNGTLKLSDVQKEVD
                                                Ig7
O. vulgaris       586 DGDYTCTAANSKGEGMSRNVLVRILEPPTITPFS-FQKQTQAGRRVTVICVISSGELPIV
O. bimaculoides     1 ------------------------------------------------------------
L. gigantea        50 AGRYTCQAKNHEGLGMTQSVYVTVVEPPVVDEFS-FGYR-KEGDRIMISCVVSSGDLPIT
C. gigas          571 VGEYTCTAKNSNGQGSYRHVYVNVVEPPVIDAFS-FENK-KLGDRIVVTCAVKTGDQPLH
D. melanogaster   584 AGTYTCMAQNKQKQTSRRNVEIQVLVPPKIMPIQAMTNMLREGMRAAISCQILEGDLPVS
S. purpuratus       1 ------------------------------------------------------------
D. rerio          570 EGEYSCHVQVQPQLFKNQSVHVTVKVPPFIQPFE-FPRY-SIGHRVFVPCVVRSGDLPIS
M. musculus       570 EGEYTCNVLVQPQLSTSQSVHVTVKVPPFIQPFE-FPRF-SIGQRVFIPCVVVSGDLPIT
H. sapiens        570 EGEYTCNVLVQPQLSTSQSVHVTVKVPPFIQPFE-FPRF-SIGQRVFIPCVVVSGDLPIT

O. vulgaris       645 VGWLRNGQVIPPDLGITVKQTIQYNSLLFIKEVSPRHNGNYTCYANNSVGYDSYTASLVV
O. bimaculoides     1 ------------------------------------------------------------
L. gigantea       108 IGWYKDNNPIPHDLGVVIQTIGPFSSLLSISDVSPRHNGNYTCYASNAAAYTNYTAPLHV
C. gigas          629 IVWTKDGEVIPPDLGIQVQKLNDFITMLSIGDANPKHNGNYTCQASNAAATTNYTATLRV
D. melanogaster   644 FRWERNGKPLIGTGNEVFRRLDEYSASLVIEHISSDHSGNYTCIASNVAGTERFTVPLTV
S. purpuratus       1 --------------------------------------------------------MVGI
D. rerio          628 ITWEKDGKSINASLGVTIDNI-DFTSSLRISNLQRVHNGTYTCIAQNDAAVVKYQSQLIV
M. musculus       628 ITWQKDGRPIPASLGVTIDNI-DFTSSLRISNLSLMHNGNYTCIARNEAAAVEHQSQLIV
H. sapiens        628 ITWQKDGRPIPGSLGVTIDNI-DFTSSLRISNLSLMHNGNYTCIARNEAAAVEHQSQLIV
```

```
                           Ig8
O. vulgaris        705 YVPPRWIVEPKDSFAVLGKSVQLHCKTSGTPTPSVIWQKAKGNIL-GNYVDMNYTQSTST
O. bimaculoides      1 ---------------------------------------------------MNYTQSTSS
L. gigantea        168 QVPPRWVIKPQNSFVVLHQSVQLDCQTAGTPTPVITWKKAADTMSPDTYETIFFD-----
C. gigas           689 DVPPRWVVEPGDSFVILKNSVSLDCLTTGSPKPTIQWKKAIGKNP-GHYQTIRYVANDNA
D. melanogaster    704 NVPPKWILEPKDSSAQAGADVILHCQSSGYPTPTITWKKAIGPTP-GEYKDFLYE-----
S. purpuratus        5 QMPPRFVVEPHNTAVVLNHTVVIACMANGKPNPDVQWKKAPGPITTTNFRDLP-------
D. rerio           687 RVPPRFKVQPQDQDGIYGKSVILNCSADGEPRPTIEWKYSKGAGV-PQFQPIALN-----
M. musculus        687 RVPPKFVVQPRDQDGIYGKAVILNCSAEGYPVPTIVWKFSKGAGV-PQFQPIALN-----
H. sapiens         687 RVPPKFVVQPRDQDGIYGKAVILNCSAEGYPVPTIVWKFSKGAGV-PQFQPIALN-----

                                                                       Ig9
O. vulgaris        764 SQTGDTRMQLLENGTLLINSAEETDHGYYLCRASNGIGYPLSRVAQLTVHIPARYEDDSQ
O. bimaculoides     10 SQTGDARMQLLENGTLLINSAEETDHGYYLCRASNGIGYPLSRVAQLTVHIPARYEDDGQ
L. gigantea        223 --PEDQNMQLMENGTLFIKNARESEHGHYLCQASNGIGYPISHRVFLTVRVPARFDA-PQ
C. gigas           748 ---TTASKQLFSNGTLVIRNAKEEDHGYYLCHSMNNVGPGISKVIFLRVHIPARFDE-VE
D. melanogaster    758 -----PTVQLFPNGTIFFKKISKESQGHFLCEAKNNIGSGVSKVIFLKVNVPAHFQT-KT
S. purpuratus       58 ---DDPRFEMQENGSLVIREARVEDAGYYLCHISNSVGMESKTAT-LT------------
D. rerio           741 ---SGFRVQLLGNGSLLIKHVLEEDAGYYLCKVSNDVGADVSKSMYLNVKIPAMITS-YP
M. musculus        741 ---G--RIQVLSNGSLLIKHVVEEDSGYYLCKVSNDVGADVSKSMYLTVKIPAMITS-YP
H. sapiens         741 ---G--RIQVLSNGSLLIKHVVEEDSGYYLCKVSNDVGADVSKSMYLTVKIPAMITS-YP

O. vulgaris        824 KNYTVKKSENSTMECKASGDEPIFVTWRFGSDT-----INSQTNSRLK----VENIGTIS
O. bimaculoides     70 KNYTVKKSENSTMECKASGDEPIFVTWRFGSDT-----INSQTNSRLK----VENIGTIS
L. gigantea        280 KNYTVLNNHNITLECQGIGDKPISITWSFKGSP-----ISTNGNKRAKVVSHDTPRGKLS
C. gigas           804 KNYTVIKGQNKTMDCQAIGDQPLSVTWSFNAQT-----ISTAGMTRRLITTTQTSRGKLS
D. melanogaster    812 KQISVAKGKQVHVQCNVQGDNPIDFKWKIQATQQ---YLDESLDSRYTIRDQVLDDGMVS
S. purpuratus      102 ----------------------VF------------------------------------
D. rerio           797 NNSLATKGEKIEMSCKAHGEKPIMVRWEKEVEKEKQSHMINPDMWRHTVTVKNVGDEVVS
M. musculus        795 NTTLATQGQRKEMSCTAHGEKPIIVRWEKE------DRIINPEMARYLVSTKEVGEEVIS
H. sapiens         795 NTTLATQGQKKEMSCTAHGEKPIIVRWEKE------DRIINPEMARYLVSTKEVGEEVIS

                                                       FN1
O. vulgaris        875 RLHVTGAVREDSGIYVCNAENKYGYGETTRRLIVVEPPERPVHLKLENRASRSVNISWEE
O. bimaculoides    121 RLHVTGAVREDSGIYVCNAENKYGYGETTRRLIVVEPPERPLHLKLESRASRSMNISWDE
L. gigantea        335 QLSLQPANRSDSGFYVCNAKNKFGHDDLMIQLVVIEPPEPPKEVRIINTTSRAITLSWKP
C. gigas           859 ALTIRPAERGDTGFYVCTAKNKFGNAVLAMRLVVLEQPESPQNLTLVKKTSRIIKVKWQP
D. melanogaster    869 ELGISHTYRQDTGIYICQASNAFGQDEMSIQLIVQEVPEQPKNLRINSQQSRSLQLTWSQ
S. purpuratus      104 ------------------------------------------------------------
D. rerio           857 TLQIYPTMREDSGFFSCHAINSYGEDRGILQLTVQEPPEPPK-VEIREVKERTIALRWTM
M. musculus        849 TLQILPTVREDSGFFSCHAINSYGEDRGIIQLTVQEPPDPPE-IEIKDVKARTITLRWTM
H. sapiens         849 TLQILPTVREDSGFFSCHAINSYGEDRGIIQLTVQEPPDPPE-IEIKDVKARTITLRWTM

O. vulgaris        935 PYNGNSYITSYVLQFKNES------DIWKPAPSNVTVIATDDIPAGSISGLHPSYKYQIR
O. bimaculoides    181 PYNGNSYITSYVLQFKNES------DIWKPAPSNVTVIATDAIPAGSISGLHPSYKYQIR
L. gigantea        395 PYDGNNPIIAYTVQFRNNS------GVWQGTLANVTVPA--GQHSVTLEELKPSYIYNIR
C. gigas           919 PYDGNSPILFYSVQYKEQK------AVWQGVIPNVTVSS--DQLIASISDLHPAYAYEVR
D. melanogaster    929 PFAGNSPIEEYHIYYKQISDFFSPSDIWQNAEH-LTI--AGAQTVINIQQLRPAKAYHIR
S. purpuratus      104 -------------------------DAWQTGLPQEHVPADLIGYQYSIKHLHPAYTYHVR
D. rerio           916 GFDGNSLITGYDIECKNKT------ETWERARRTRDVSP--TLNQATIIELHPSSTYNIR
M. musculus        908 GFDGNSPITGYDIECKNKS------DSWDSAQRTKDVSP--QLNSATIIDIHPSSTYSIR
H. sapiens         908 GFDGNSPITGYDIECKNKS------DSWDSAQRTKDVSP--QLNSATIIDIHPSSTYSIR

                                                FN2
O. vulgaris        989 ALAINAIGTGSPSTVITVSTDEEVPSGPPTDVRVQASGSTSLKVTWRAPLYQEQNGKILG
O. bimaculoides    235 ALAINAIGIGSPSTVITVSTDEEVPSGPPTDVRVQASGSTSLKVTWRAPLYQEQNGKILG
L. gigantea        447 IMANNSVGYSVPSPDQVVTTEEEAPTGPPGNVVAKAIGSQSLQVTWESPADGYVNGKILG
C. gigas           971 VLANNSIGYGKASGSLIIKLDEEKPSGPPTEVNVKAIGSESLWISWMPPLPDHQNGEILG
D. melanogaster    986 MSAENKLGASEFSEVVQVTTLEEVPSGPPLAVRAEPKSSTEIFVTWDAPERDHWNGILLG
S. purpuratus      139 IFAFNAIGISDPSKEILFDTEEEAPSGIPLNIEIEALSSQSIQITWRQPRADLQNGFLLG
D. rerio           968 MFAKNHIGDSEPSNELTVTTDEADPEGPPQDVTLEAITSQSIKVTWKAPLKHLQNGVIRG
M. musculus        960 MYAKNRIGKSEPSNEITITADEAAPDGPPQEVHLEPTSSQSIRVTWKAPKKHLQNGIIRG
H. sapiens         960 MYAKNRIGKSEPSNEITITADEAAPDGPPQEVHLEPISSQSIRVTWKAPKKHLQNGIIRG
```

```
O. vulgaris      1049 YYIGYKRH--------NSSDSYVYRKKLTENNSD-LEQQIKDLLKFTRYDLHVRAYNSKG
O. bimaculoides   295 YYIGYKRH--------NSSDSYVYRKKLTENNSD-LEQQIKDLLKFTRYDLHVRAYNSKG
L. gigantea       507 YYIGYKET--------NSPSHFIYITQTVGEDFQ-AKHTIHNLKKFTEYTVHVKAYNAKG
C. gigas         1031 YYIGYKEK--------DSQSRFIYITKSIDGEFL-PEVDIHNLEKFTEYTVHVQAYNQLG
D. melanogaster  1046 YYVGYQMSLTPEDKEVNPTQGFSFKTVEVRSHFG-GETVLANLNKFTQYHVIVQAYTSQG
S. purpuratus     199 YHIQYCDP----------DSPFQSRIVPVETNYI-ESRTLTNLKKFTRYTIQLRAYNRVN
D. rerio         1028 YQVCHREH--------STNGSHQFVCISVEATGETESLSLNNLKKFAEYEVVVQASNSAG
M. musculus      1020 YQIGYREY--------STGGNFQENIISIDTTGDSEVYTLDNLNKFTQYGLVVQACNRAG
H. sapiens       1020 YQIGYREY--------STGGNFQENIISVDTSGDSEVYTLDNLNKFTQYGLVVQACNRAG

O. vulgaris      1100 LGPITDDVTAFTLED---------------------------------------------
O. bimaculoides   346 LGPITDDVTAFTLED---------------------------------------------
L. gigantea       558 ISPASDNIQVFTLEDADVECVLNSVQTCIIERSVDSMIPEGAELTNGGNKSSVIMERSCQ
C. gigas         1082 RGPPSPDAQVFTLED---------------------------------------------
D. melanogaster  1105 SGPPSKEIAVQTMED---------------------------------------------
S. purpuratus     248 VGPWSPQQFVTTLED---------------------------------------------
D. rerio         1080 PGPASSEVRATTMED---------------------------------------------
M. musculus      1072 TGPSSQEIITTTLED---------------------------------------------
H. sapiens       1072 TGPSSQEIITTTLED---------------------------------------------

                              FN3
O. vulgaris      1115 ------VPSLPPQEVRIRDINPDNIRISWVEPPYYTVQGVLQGYKVLYKEVRADEDETEA
O. bimaculoides   361 ------VPSLPPQEVRIRDINPDNIRISWVEPPYYTVQGVLQGYKVLYKEVRADEDETEA
L. gigantea       618 VVHQPAVPSQPPQNVQANPVSSVAIRVLYAPPPLYILHGILLGYKVLYKPVRNDEDESDA
C. gigas         1097 ------VPSQPPQGVQATAINSRSIKVVWSPPPLFTLHGILQGYKIYYKPVRFDEDESDS
D. melanogaster  1120 ------VPSSPPESPQCDVLGSTSIYITWSPPDIDGQNGKIKGYKVFYISVDEL-YETDP
S. purpuratus     263 ------VPSEAPHDVSARSVGSTSIMVFWNAPDQVSLNGILQGYRVYYRPIRENEDELDY
D. rerio         1095 ------VPSRAPEKVVATAASPESISLSWQTPAREALNGVLQGFRIIYWANLPDGELGEI
M. musculus      1087 ------VPSYPPENVQAIATSPESISISWSTLSKEALNGILQGFRVIYWANLIDGELGEI
H. sapiens       1087 ------VPSYPPENVQAIATSPESISISWSTLSKEALNGILQGFRVIYWANLMDGELGEI

                                                                        FN4
O. vulgaris      1169 SDVTTSKLETVVTDLKKYTNYSIQVLAYTIMGEGVRSEPIYVMTREDVPSQPPNVKVITE
O. bimaculoides   415 SYVTTSKLETVVPDLKKYTNYSIQVLAYTIMGEGVRSEPIDIRTREDVPSQPPNVKVITE
L. gigantea       678 SFVTTDSMEAMLYKLEKYTNYSIQVLAYTRKGEGVRSEPIYVTTQEDLPESPSDIKSLPV
C. gigas         1151 NAVVSSELEATITGLSKYTNYSLQVLAFTRKGEGVRCEPLFVLTQQDAPERPADIKALPV
D. melanogaster  1173 EVVKSTNQYVTIENLRKYTNYTVWVLAYTKVGDGMKTKPFYCRTHEDVPSAPQAIKAIPA
S. purpuratus     317 QIYETTELFAELHNLQKFTNYSLSVVAHTRVGEGVRSEEKIVRTEQDVPEAPADIKAHAA
D. rerio         1149 RNVTTHKAPLELEGLEKYTNYSIQVLAFTRAGDGVRSDQIYIRTKEDVPGPPGGVKAAAA
M. musculus      1141 KNVTTTQPSLELDGLEKYTNYSIQVLAFTRAGDGVRSEQIFTRTKEDVPGPPAGVKAAAA
H. sapiens       1141 KNITTTQPSLELDGLEKYTNYSIQVLAFTRAGDGVRSEQIFTRTKEDVPGPPAGVKAAAA

O. vulgaris      1229 NRNIVLLLWEPPVHQNGILIKYIIRYSNSSRGPPNKKSPKLEIKARRTSYMISRLLEGQE
O. bimaculoides   475 NRNIVLLLWEPPVHQNGILIKYIIRYSNSSRGPPNKKSPKLEIKARRTSYMISRLLEGQE
L. gigantea       738 NSSSIIVAWQLPEHPNGEITEFTLYVLNNTADS-KNLEDKVKLPADTSSYLVSNLQVGHQ
C. gigas         1211 SNTSVMASWKPPLHSNGILTKYNVYVYNSTS---TELMSKLELPPVKTSYYVNNLSLNEE
D. melanogaster  1233 SSSKIIISWLPPDLPNGDITGYTFYMSMLEGGREEGTHKRL-LGPFVEMHETVRTQESAT
S. purpuratus     377 SPTSIMVSWLPPLYLNGIITHYHLNIQYREG--ENEVRQEKELDPTTYYYLVEDLQTDYE
D. rerio         1209 SSSVVYVSWLPPLKLNGVIRKYTVFCSSTSP----TVVSEFEVAPDEFLHRVHNLSRNRK
M. musculus      1201 SASMVFVSWLPPLKLNGIIRKYTVFCSHPYP----TVISEFEASPDSFSYRIPNLSRNRQ
H. sapiens       1201 SASMVFVSWLPPLKLNGIIRKYTVFCSHPYP----TVISEFEASPDSFSYRIPNLSRNRQ

                                                 Ig10
O. vulgaris      1289 YLFTVSACTYGGESIPSDVVRATPTDKVPARITNFSREQTLYWKTNSILKCNARGNPIPL
O. bimaculoides   535 YMFTVSACTYVGESLPSDVVRATPTDKVPARITNFSREKTLYWKTNSILKCNARGNPLPL
L. gigantea       797 YGFRITASTMIGEGKPTALILASPQENVPARIASFSTKLIVPWHESITFPCLAVGDPEPS
C. gigas         1268 VRFEVSANTIIGEGERIHSVTTAPQEKVAARIASFSAVMLIPWQHTLTLQCLAVGDPAPI
D. melanogaster  1292 YQFWLTASTKMGEGEKTQVVTVPPNNKVPARIVSFSQRIVTPWKEHIELPCRKVGAPAPV
S. purpuratus     435 YDFWVKASTIAGQGESSRIATEIPMSRVPARIMSFSTTLMTPWKRGVTLDCLPVGDPVLT
D. rerio         1265 YNIWVMAVTAAGRGNSSDVITVEPLAKAPAKILTFSGTVTTPWMRDVVLPCRAVGDPPPA
M. musculus      1257 YSVWVVAVTSAGRGNSSEIITVEPLAKAPARILTFSGTVTTPWMKDIVLPCKAVGDPSPA
H. sapiens       1257 YSVWVVAVTSAGRGNSSEIITVEPLAKAPARILTFSGTVTTPWMKDIVLPCKAVGDPSPA
```

```
O. vulgaris      1349 ISWIMKDK----PVLLSKTVQKLPSGSLNLKSVQFTDAANYTCRAENIYGIEE-IVISVK
O. bimaculoides   595 ISWIMNDK----PVLLSNTVQKLPSGSLNLKSVQFTDTANYTCRAENIYGIEE-IVISVK
L. gigantea       857 VEWKLRGH----TLKQSERVKVLENGSLYFDDVIGTDAANYSCHAKNKHGFDD-ITYILQ
C. gigas         1328 IKWKIRGR----PLIVNERLQILKNGSLHINGVLGSDAANYSCRSENVYGSDE-INYAIS
D. melanogaster  1352 TIWRQDGH----NMETSARKTIAKNGTLYMKECQASDAGNYTCSVENTWGKDE-IVYNIV
S. purpuratus     495 VEWKKNR--LPNVLKPDGRIDILENGSLVIQSVQSTDAGNYTCRAINEYGSSDSITVRVQ
D. rerio         1325 IKWLKES-GSPAPAVIDGRRSIHGNGSFIIKTVKAEDSGYYTCVASNNYGTDE-IILNLQ
M. musculus      1317 VKWMKDSNGTPSLVTIDGRRSIFSNGSFIIRTVKAEDSGYYSCVANNNWGSDE-IILNLQ
H. sapiens       1317 VKWMKDSNGTPSLVTIDGRRSIFSNGSFIIRTVKAEDSGYYSCIANNNWGSDE-IILNLQ

                             FN5
O. vulgaris      1404 VIVKKKDPVVPREPKLMLASSTASTIQVNWNSRHNGGSKIKGFYVKFKKEH-GEWQSLKA
O. bimaculoides   650 VIVKQKNPVVPLEPILVLASSTSSTIQVNWKSRHNGGSKIKGFYVKFKKEH-GEWQSLKA
L. gigantea       912 VQA------PPQPARLYLALTTSSTIQVNWLSGSNGGSPIQGFVLNYKKEH-GSWQDIRL
C. gigas         1383 VQA------PPKPPSLYVVATTTSTIQMNWRSGSNGGSPIQGFVIHFKKDH-EVWQKIHA
D. melanogaster  1407 VKV------PPEAPNLTVINAYTDSLLLEWMDNSHGGSPILGYVINYKRDN-GDWEELQV
S. purpuratus     553 VLA------PPNPPQLTIGSTSNNAIKVNWRSTGNGGSSIVAFQLLYQRTHEQTWERFDV
D. rerio         1383 VQV------PPDQPRLTVTKTTTTSITVTWTPGDNGGSSIRGYILQYSEDNSEKWGSISI
M. musculus      1376 VQV------PPDQPRLTVSKTTSSSITLSWLPGDNGGSSIRGYILQYSEDNSEQWGSFPI
H. sapiens       1376 VQV------PPDQPRLTVSKTTSSSITLSWLPGDNGGSSIRGYILQYSEDNSEQWGSFPI

                                                                   FN6
O. vulgaris      1463 DSISRTYMFKGLQCGTGYRFIINSFNIIGVSGDSTQINAKTNGSSPVMPAQELILKSVNS
O. bimaculoides   709 DSISRTYIFKGLQCGTGYRFIVNSFNIIGVSGDSTQINAKTNGS----------------
L. gigantea       965 GPQNRTYIAANLLCGTLYKFNIRAFNRLGDSLDSDIVTAKTNGSVPIMPPQSMLVKEVNA
C. gigas         1436 GPTNRTQTVTGLLCGTTYKFYINALNRLGYSPDSDIMSVKTNGSTPIMPPQSMLLKVINV
D. melanogaster  1460 DSKTTSHLLTNLWCGTRYQLYITAYNKIGTGLPCDIVNSYTKGNPPVQPKHSQMI-TNNS
S. purpuratus     607 NASLRSFRATDLLCGTGYRFYITARNKLGVGKPSDILSVSTQGEIPIPPSTDKIVPFTNA
D. rerio         1437 SPSERSYRLENLNCGTWYKFTLTAQNAVGPGRISEIIEAKTHGKEPQFSKEQELFTSING
M. musculus      1430 SPSERSYRLENLKCGTWYKFTLTAQNGVGPGRISEIIEAKTLGKEPQFSKEQELFASINT
H. sapiens       1430 SPSERSYRLENLKCGTWYKFTLTAQNGVGPGRISEIIEAKTLGKEPQFSKEQELFASINT

O. vulgaris      1523 TFVQLDLSAWSSGGCPIKFYTVKYRMNGHDEWVEVSNNVDGNMTSFTVRDLRPATWYWMK
O. bimaculoides   753 ---------SWSSGGCPIKFYTVKYRMNGHTEWVEVSNNVDGNMTSFTVRDLRSATWYWMK
L. gigantea      1025 TSVILDLRTWRTSGCPIRFFSVKYQVFGDNIWTFVSNSMRYNTTLFTVQDLHPATWYKMV
C. gigas         1496 TSVDLDLNTWITSGCPIRFFSVQYKVWGDTAWTEVSNNIQSNKTIHTVEDLHPATWYVMK
D. melanogaster  1519 TSVTCWLDSWGDGGCGILYFMIESRVYGRSSWAVVSNHIPPTERIYTVSDLVPGTKYQLK
S. purpuratus     667 SSITLVLASWQSGGCPIESYSIQYQVLGVDDWENIANNLPGNTRDYTVDDLRPLTWYQFR
D. rerio         1497 TSVKLNLIGWNDGGCPITSFTLEYRPLESPVWTKAKRT--SLSKSYNLLDLQEATWYELQ
M. musculus      1490 TRVRLNLIGWNDGGCPITSFTLEYRPFGTTVWTTAQRT--SLSKSYILYDLQEATWYELQ
H. sapiens       1490 TRVRLNLIGWNDGGCPITSFTLEYRPFGTTVWTTAQRT--SLSKSYILYDLQEATWYELQ

O. vulgaris      1583 LTAHNEAGSTESLFNFGTLTYSGS------------------------------------
O. bimaculoides   805 LTAHNEAGSTESLFNFGTLTYSGS------------------------------------
L. gigantea      1085 VTAHSDAGSSESKLTFATLTYTGTLSNNFLIKYSINSSVDLPSVINKQQNPCIINNVMRS
C. gigas         1556 VIAHSDAGSTESELKFATLTYHGH------------------------------------
D. melanogaster  1579 VTAHNNAGSTTAIYNFTTLSTQGVIYNND-------------------------------
S. purpuratus     727 VTAVNTAGPSVYPFRVSTVAFSGS------------------------------------
D. rerio         1555 MKVYNSAGLAEKRVKFATLSYDGS------------------------------------
M. musculus      1548 MRVCNSAGCAEKQANFATLNYDGS------------------------------------
H. sapiens       1548 MRVCNSAGCAEKQANFATLNYDGS------------------------------------

O. vulgaris      1607 -TIRPLFVV---HRKEGKFYEKIYVMLPLCTGIILILVAAVAV--LLWCRRRSERLRFKE
O. bimaculoides   829 -TIRPLFVV---HRKEGKFYEKIYVMLPLCTGIVLILVAAVAV--LLWCRRRSERLRFKE
L. gigantea      1145 GTILPIQVI---HKEESEFYEKITIMVPLCAGLVSLSVAIVGS--ILFCRRRRERLRYK-
C. gigas         1580 -TITPLFIT---KKEESNFYEKTYIMIPLCFGIVAFLILIIAV--LLYMRRRRELIQMK-
D. melanogaster  1608 ---HSTPVS---HLSDLPFYANFKLLLPICFSLLMLLAL-IGA--ALFLRKRKLASQARL
S. purpuratus     751 GTAPPIKQS---TSRPLAFYEDPRLFGPILGILTLFVILGFISYFIHGCRRRRKKSVKS-
D. rerio         1579 -TIPPLVKTLVKDPVKKTSNEGMKMMVTISCILVGMVLLFVLL---MVLRRRRREQRLKR
M. musculus      1572 -TIPPLIKSVVQSEEGLTTNEGLKILVTISCILVGVLLLFVLL---LVVRRRRREQRLKR
H. sapiens       1572 -TIPPLIKSVVQNEEGLTTNEGLKMLVTISCILVGVLLLFVLL---LVVRRRRREQRLKR
```

```
O. vulgaris      1661 ------------------------------------------------------------
O. bimaculoides   883 LFRNGSKALVQEMIALKINQGVGQNQHQNPRTKVRKLSSELQNLTQLFLDTCKKLEVKKT
L. gigantea      1199 ------------------------------------------------------------
C. gigas         1633 ------------------------------------------------------------
D. melanogaster  1659 ASSSM-------------------------------------------------------
S. purpuratus     807 ---QHVVVETLSGRESNG------------------------------------------
D. rerio         1635 LRDAKSLAEMLMSKNTRP------------------------------------------
M. musculus      1628 LRDAKSLAEMLMSKNTRT------------------------------------------
H. sapiens       1628 LRDAKSLAEMLMSKNTRT------------------------------------------

O. vulgaris      1661 ------------------------------------------------TASNLR--RDIT
O. bimaculoides   943 DRKKSAEVKSGERDGPSSGPRLPIHRQGRLASNQSRTSVACAEFHFPETASNLR--RDIT
L. gigantea      1199 -----------------------------------------------EHASNLR--RDIT
C. gigas         1633 -----------------------------------------------ESASNLR--RDIT
D. melanogaster  1664 ------------------------------------------------------------
S. purpuratus     822 --------------------------------------------NSPSQQNNRPTVHLP
D. rerio         1653 ------------------------------------------SDTMNKQQQTLRMHIDIP
M. musculus      1646 ------------------------------------------SDTLSKQQQTLRMHIDIP
H. sapiens       1646 ------------------------------------------SDTLSKQQQTLRMHIDIP

O. vulgaris      1671 ------AETSLMNDLDKRLN-----LDMDCGGGMPDSFCKRNLSLLTSATLPELMANNGR
O. bimaculoides  1001 ------AETSLMNDLDKRLN-----LDMDCGGGMPDSFCKRNLSLLTSATLPELMANNGR
L. gigantea      1210 ------AETSLMNDLDKRLN-----VDLDSSTSTLPEFSKRNVNLLISLHSDDNINGNS-
C. gigas         1644 ------AETSLMNDLDKRFN-----FDFDGG--NTEPYGKRNVNLLISFNSDENLTNNS-
D. melanogaster  1664 ------SESPSLANLQNKQNRDQQYLAVRCNPGTSAPRG---------------------
S. purpuratus     838 -----PEREPFLGAY--------------------------------------------E
D. rerio         1671 RAQLLIEERDTMETVDDRST-----------------------VLLTDN-----------
M. musculus      1664 RAQLLIEERDTMETIDDRST-----------------------VLLTDA-----------
H. sapiens       1664 RAQLLIEERDTMETIDDRST-----------------------VLLTDA-----------

O. vulgaris      1720 ANGSAGSEHTSWLFHTAYPSWDGGKNSGDISYAPNRAGIAVHPWSSPYAATLSSRYVSNT
O. bimaculoides  1050 ANGSAGSEHTSWLFHTAYPSWDGGKNSGDISYAPNRAGIAVHPWSSPYAATLSTRYASNT
L. gigantea      1258 ---------QSWLIDSSK----TNSENGSFSRSEDEGN--INP----YATFNELKAVVNT
C. gigas         1690 ---------PTWLNNGSSK---TNSDNGSISRSEDDGN--INP----YATFNQMKQVIME
D. melanogaster  1697 --------------------SNSNDSGSFGKAEGNEY--IE-DICPYATFQLNKQTYSE
S. purpuratus     849 RAETIAVRQTPWIFNADE-------EEGDYASEPRRHRG----LADPYATFDYHDGSIYP
D. rerio         1697 ----------------------------DFGETAKQKSATVT-HSVHYQSLS--------
M. musculus      1690 ----------------------------DFGEAAKQKSLTVT-HTVHYQSVS--------
H. sapiens       1690 ----------------------------DFGEAAKQKSLTVT-HTVHYQSVS--------

O. vulgaris      1780 APKTATSYKTKPYSSNCYDTTAPYASVSVDAL---------DDTNPYAAINQL-----SI
O. bimaculoides  1110 APKTVTSYKTKPYSSNVYDTTAPYASVSVDAL---------DDTNPYATINQL-----SI
L. gigantea      1299 GKRVEMMQK----------SSESEDEIALQKAANQREGLMATPCEPYVPFFHSKGATGTV
C. gigas         1732 K--EQQSE---------RDKSPDLDDISLQKL--EAQKAMISPSEPYVPFFHAKGDNGDV
D. melanogaster  1733 S----------SYSGNVYS--GPYHSVRGSFV--------YHDVKPE--SYHS-------
S. purpuratus     898 SMS-------------TFRSDGSSASLS-------DCGLMIHDERLYDI-----------
D. rerio         1720 ------------------QATGPLVDVSD---------------VRPGTSKSS---------
M. musculus      1713 ------------------QATGPLVDVSD---------------ARPGT------------
H. sapiens       1713 ------------------QATGPLVDVSD---------------ARPGT------------

O. vulgaris      1826 TINPSKP--------GLHANSQSSDEDTVGLEK---------AA----AQRQ--TRGPPL
O. bimaculoides  1156 TINPSKP--------GLHANSQSSDEDTVGLEK---------AA----AQRQ--SVAYIF
L. gigantea      1349 PRKPPPLPKTRPPRRTRHEEVKKEGYDNHGVLL---------SPRKYASADQIHALFTQA
C. gigas         1779 AKDPHPP--------LIP-LKTTEGYDNQGLIL---------SPRKYASADQIHALFTQV
D. melanogaster 1764 –KEPEYTKVRRKVGRLRDPHSESQEYDTLGSESDNDVSARALNSAKYRAQRDTQDETS SS
S. purpuratus     927 --PPEE------------------------------------------------------
D. rerio         1740 LSNPT---SRRTAK------------------------AGPAARNRYASQWTLNRPHPPV
M. musculus      1729 --NPT---TRRNAK------------------------AGPTARNRYASQWTLNRPHPTI
H. sapiens       1729 --NPT---TRRNAK------------------------AGPTARNRYASQWTLNRPHPTI
```

```
O. vulgaris       1863 KMR-------PRMPYPNSSSICKPPRK-KKEGYENKGGQRHSVISSVTTVSSSRDE-LLE
O. bimaculoides   1193 HTF-----------------V-------VQPDVALAGSQRHSVISSVTTVSSSRDE-LLE
L. gigantea       1400 PPR-------PPSC-----------HK-SGSGSSDKGSQRHSLISSVTTVSSSRDE-LLE
C. gigas          1821 PAR-------PHSSYS-------KSKK-CPSSSDEKGSQRQSIISSVTTVSSSRDE-LLE
D. melanogaster   1823 SET-------TPTSMT---RKSKPPFAARKGGKPGTSGKRHVRSSS--GYSSHNEETTFR
S. purpuratus      931 --------------------------------IAHRGNKRPSNGSLSTTNESSNREELER
D. rerio          1773 SVHTLSTDWRLGTPRV-AGSVDKESDSYSVSPSQDTDRARSSMVST-ESA-SSTYEELAR
M. musculus       1760 SAHTLTTDWRLPTPRA-TGSVDKESDSYSVSPSQDTDRARSSMVST-ESA-SSTYEELAR
H. sapiens        1760 SAHTLTTDWRLPTPRA-AGSVDKESDSYSVSPSQDTDRARSSMVST-ESA-SSTYEELAR

O. vulgaris       1914 AYENAKRTQRAQPIVYETHP------------------DFSSQPTDSSSATEPGIREFT
O. bimaculoides   1228 AYENAKRVQRAQPIVYETHP------------------DFSSQPTDSSSATEPGIREFT
L. gigantea       1440 ALENAKKN-PPPPVLFESQN------------------DDSSQPTDSSIATEPGIREFT
C. gigas          1865 ALENAKHN-PPPPVVYESQP------------------ESSSQPTDSSVGTEPGIVKFT
D. melanogaster   1871 TL-------------YV-------------------------------------------
S. purpuratus      959 AYSTGMRIEA---AQFELIQCANKKREDYSPASSSSRSHVSRDHPSVINSDVEHGIRNFT
D. rerio          1830 AYEHAKMEEQLRHAKFTITECFISDTSSEQM----TAGTNDYTDSLTSSTPSESGICRFT
M. musculus       1817 AYEHAKMEEQLRHAKFTITECFISDTSSEQL----TAGTNEYTDSLTSSTPSESGICRFT
H. sapiens        1817 AYEHAKMEEQLRHAKFTITECFISDTSSEQL----TAGTNEYTDSLTSSTPSESGICRFT

O. vulgaris       1955 QSPPKPDERREAS-----------------------CEVPPYEQRR---HLKEFDAESDT
O. bimaculoides   1269 QSPPKPDERREAS-----------------------CEVPPYEQRR---HLKEFDAESDT
L. gigantea       1480 QSPPKPNEQREAS-----------------------CEVPSYEQQKRR-RQHRKEMESDT
C. gigas          1905 QSPPKPNEQREAS-----------------------CEVHYTSDMKPTIKRSRKEAESDT
D. melanogaster        ------------------------------------------------------------
S. purpuratus     1016 ASPPMPTVPEANHYAPRRYVHTLEGARPRDRARMSLTTSPSLVRSSEESSDVDFEMESSE
D. rerio          1886 ASPPKPQDVCRVINMAVPKAHRPGG---------ELVHLPPYLRMDFLLNRG---VSGTS
M. musculus       1873 ASPPKPQDGGRVVNMAVPKAHRPG----------DLIHLPPYLRMDFLLNRG---APGTS
H. sapiens        1873 ASPPKPQDGGRVMNMAVPKAHRPG----------DLIHLPPYLRMDFLLNRG---GPGTS

O. vulgaris       1989 TECEMAHTRERSPPRR-IRGRHK--GKQKGQV-VSKRNLGI-IPRTHSRTST-----TST
O. bimaculoides   1303 TECEMAHTRERSPPRR-IRGRHK--GKQKGQV-VSKRNLGI-IPRTHSRTST-----TST
L. gigantea       1516 TECEGGELQDHSPPRRTIRGRRN--KQKPQII-SKRSLGTTF-IPRAHSRTST-----T--
C. gigas          1942 TECETNE--RPTQPRR-IKGRRN--KQRNQLT-KRQVMPSY-VPRTHSRTST-----T--
D. melanogaster        ------------------------------------------------------------
S. purpuratus     1076 ----RGSIHVY-RPSRYRSNRRVPRRVPPGSISDPERGRHLRHPKRHATSVGGVKVVHPI
D. rerio          1934 REAAMGQACLEPQKTRS--------LKRPALI----------EPTPMEVPTS--------
M. musculus       1920 RDLSLGQACLEPQKSRT--------LKRPTVL----------EPTPMEASSS-----TSS
H. sapiens        1920 RDLSLGQACLEPQKSRT--------LKRPTVL----------EPIPMEAASS-----ASS

O. vulgaris       2039 NSEEVTYAFCERDYPKPGSP---VESYNPYTCDTHPCSDVELDADNLLNKSRTSIRSPIF
O. bimaculoides   1353 NSEEVTYAFCERDYPKPGSP---VESYNPYTCDTHPCSDVELDADNLLNKSRSSIRSPIF
L. gigantea       1566 SSEEVTYTFGGRESPRSNSP---SEGYLPYPRDGYGYPETDVVTR-----TR-----RDN
C. gigas          1988 SSEEVTYTFQGRQSP--HSP---PRGYSSYLPDSQL---------------------PES
D. melanogaster        ------------------------------------------------------------
S. purpuratus     1131 HPETVRYHIPSEIREEGESPKKDPRSCKPMMITSYHSPPPP--------------PPPSL
D. rerio          1968 --RDVQQWQSGTAST------------LPQREA-----GTDLAQAAKISTS----QESLL
M. musculus       1957 TREGQQSWQQGAVAT------------LPQRE------GAELGQAAKMSSS----QESLL
H. sapiens        1957 TREG-QSWQPGAVAT------------LPQRE------GAELGQAAKMSSS----QESLL

O. vulgaris       2096 FRNNAQNGRNTPHSRM-RYDVNLDIPGTEECKPLVMTLNQAQTISPTIEQ---DNISILD
O. bimaculoides   1410 FRNNAQNGRNTPHSRI-RYDVNLDIPGTEECKPLVMTLNQAQTTSPTIEQ---DNISILD
L. gigantea       1613 KSRGRGTGRGTPHGKR-RYDVSIQTPGTDENKPLVMALAQPALTSPAEEDE---HVSLLD
C. gigas          2022 DKTPMRRGRRTPHAKV-KYDLSLQTPGTDESRPLVMAVAQPSMSSPNEEDEDSATVSLLN
D. melanogaster        ------------------------------------------------------------
S. purpuratus     1177 ----PPKRRGSPEGNEQDIEEEIEIQVIDECSK----------ASPI-GDQ---------
D. rerio          2005 DSRGHLKQTNNP------------------------------------------------
M. musculus       1995 DSRGHLKG-NNP------------------------------------------------
H. sapiens        1994 DSRGHLKG-NNP------------------------------------------------
```

```
O. vulgaris      2152 RHYRPVKDPGL-LPPESPSRITLERLNTNYTANYTIV--
O. bimaculoides  1466 RHYRPVKDPGL-LPPESPSRITLERLNTNYTANYTIV--
L. gigantea      1669 RYYDHYRCYDN-----------------YYYYYY-----
C. gigas         2081 RHYRPVEQEEGEITGQQ------KSRDKGYREDFTLV--
D. melanogaster       ---------------------------------------
S. purpuratus    1213 -----------------------------FRENISVV--
D. rerio         2017 -----------------------------YAKSYTLTRR
M. musculus      2006 -----------------------------YAKSYTLV--
H. sapiens       2005 -----------------------------YAKSYTLV--
```

Table S7: Percentage identity matrix of Ov-DSCAM.

Ov-DSCAM	Accession number	*Ov*	*Ob*	*Lg*	*Cg*	*Sp*	*Dm*	*Dr*	*Mm*	*Hs*
O. vulgaris	c34599_g6_i1[6]	100	95	46	45	34	32	33	34	34
O. bimaculoides	Ocbimv22004917	95	100	45	45	32	31	32	33	33
L. gigantea	LotgiG163888	46	45	100	57	34	36	34	35	35
C. gigas	EKC35374	45	45	57	100	36	35	34	35	35
D. melanogaster	FBgn0263219	34	32	34	36	100	34	31	33	32
S. purpuratus	SPU_003460	32	31	36	35	34	100	32	33	33
D. rerio	ENSDARG00000024865	33	32	34	34	31	32	100	79	79
M. musculus	ENSMUSG00000050272	34	33	35	35	33	33	79	100	98
H. sapiens	ENSG00000171587	34	33	35	35	32	33	79	99	100

[6] For the corresponding GenBank Accession Number refer to table S8.

Accession numbers

Table S8: Accession numbers of Ov-PCDHs and Ov-DSCAM.

Name	Accession number	
Protocadherin	c30292_g8_i3	MK_216633
Protocadherin	c36429_g1_i2	MK_216634
Protocadherin	c30659_g1_i2	MK_216635
Protocadherin	c29866_g1_i2	MK_216636
Protocadherin	c34361_g3_i2	MK_216637
Protocadherin	c36174_g3_i1	MK_216638
Protocadherin	c36706_g1_i1	MK_216639
Protocadherin	c33102_g8_i3	MK_216640
Protocadherin	c30160_g2_i2	MK_216641
Protocadherin	c33357_g1_i3	MK_216642
Protocadherin	c29866_g2_i2	MK_216643
Protocadherin	c33102_g14_i1	MK_216644
Protocadherin	c36249_g6_i1	MK_216645
Protocadherin	c36249_g4_i5	MK_216646
Protocadherin	c31878_g4_i1	MK_216647
Protocadherin	c33164_g2_i1	MK_216648
Protocadherin	c33164_g3_i1	MK_216649
Protocadherin	c32351_g9_i1	MK_216650
Protocadherin	c33402_g7_i1	MK_216651
Protocadherin	c36429_g1_i1	MK_216652
Protocadherin	c36393_g5_i2	MK_216653
Protocadherin	c33102_g3_i1	MK_216654
Protocadherin	c31573_g9_i1	MK_216655
Protocadherin	c35355_g4_i1	MK_216656
Protocadherin	c31951_g8_i3	MK_216657
Protocadherin	c29866_g5_i1	MK_216658
Protocadherin	c32474_g4_i1	MK_216659
Protocadherin	c35066_g15_i1	MK_216660
Protocadherin	c30292_g11_i1	MK_216661
Protocadherin	c30292_g10_i2	MK_216662
Protocadherin	c36667_g2_i5	MK_216663
Protocadherin	c29984_g2_i1	MK_216664
Protocadherin	c36451_g2_i1	MK_216665
Protocadherin	c30292_g8_i1	MK_216666

Name	Accession number	
Protocadherin	c34569_g11_i1	MK_216667
Protocadherin	c29866_g4_i3	MK_216668
Protocadherin	c34392_g5_i1	MK_216669
Protocadherin	c32349_g7_i2	MK_216670
Protocadherin	c30292_g7_i1	MK_216671
Protocadherin	c33760_g1_i1	MK_216672
Protocadherin	c32114_g1_i5	MK_216673
Protocadherin	c36614_g10_i1	MK_216674
Protocadherin	c33221_g2_i2	MK_216675
Protocadherin	c36451_g1_i3	MK_216676
Protocadherin	c36249_g4_i2	MK_216677
Protocadherin	c36241_g1_i1	MK_216678
Protocadherin	c30292_g9_i1	MK_216679
Protocadherin	c33221_g2_i1	MK_216680
Protocadherin	c28654_g1_i2	MK_216681
Protocadherin	c32730_g4_i1	MK_216682
Protocadherin	c27606_g2_i2	MK_216683
Protocadherin	c31207_g1_i5	MK_216684
Protocadherin	c36249_g4_i1	MK_216685
Down syndrome cell-adhesion molecule	c34599_g6_i1	MK_216686

References

Albertin, C.B., Simakov, O., Mitros, T., Wang, Z.Y., Pungor, J.R., Edsinger-Gonzales, E., Brenner, S., Ragsdale, C.W., and Rokhsar, D.S. (2015). The octopus genome and the evolution of cephalopod neural and morphological novelties. *Nature* 524(7564)**,** 220-224.

Artimo, P., Jonnalagedda, M., Arnold, K., Baratin, D., Csardi, G., De Castro, E., Duvaud, S., Flegel, V., Fortier, A., and Gasteiger, E. (2012). ExPASy: SIB bioinformatics resource portal. *Nucleic Acids Research* 40(W1)**,** W597-W603.

Finn, R.D., Coggill, P., Eberhardt, R.Y., Eddy, S.R., Mistry, J., Mitchell, A.L., Potter, S.C., Punta, M., Qureshi, M., Sangrador-Vegas, A., Salazar, G.A., Tate, J., and Bateman, A. (2016). The Pfam protein families database: towards a more sustainable future. *Nucleic Acids Research* 44(D1)**,** D279-D285. doi: 10.1093/nar/gkv1344.

Finn, R.D., Mistry, J., Schuster-Böckler, B., Griffiths-Jones, S., Hollich, V., Lassmann, T., Moxon, S., Marshall, M., Khanna, A., and Durbin, R. (2006). Pfam: clans, web tools and services. *Nucleic Acids Research* 34(suppl_1)**,** D247-D251.

Gu, Z., Eils, R., and Schlesner, M. (2016). Complex heatmaps reveal patterns and correlations in multidimensional genomic data. *Bioinformatics* 32(18)**,** 2847-2849.

Kersey, P.J., Allen, J.E., Allot, A., Barba, M., Boddu, S., Bolt, B.J., Carvalho-Silva, D., Christensen, M., Davis, P., Grabmueller, C., Kumar, N., Liu, Z., Maurel, T., Moore, B., McDowall, M.D., Maheswari, U., Naamati, G., Newman, V., Ong, C.K., Paulini, M., Pedro, H., Perry, E., Russell, M., Sparrow, H., Tapanari, E., Taylor, K., Vullo, A., Williams, G., Zadissia, A., Olson, A., Stein, J., Wei, S., Tello-Ruiz, M., Ware, D., Luciani, A., Potter, S., Finn, R.D., Urban, M., Hammond-Kosack, K.E., Bolser, D.M., De Silva, N., Howe, K.L., Langridge, N., Maslen, G., Staines, D.M., and Yates, A. (2018). Ensembl Genomes 2018: an integrated omics infrastructure for non-vertebrate species. *Nucleic Acids Research* 46**,** D802-D808. doi: 10.1093/nar/gkx1011.

Kulesza, J., Spencer, J., and Sood, A. (2017). *White Paper. Standardization of Color Palettes for Scientific Visualization.* Washington, DC, USA: The National Academies of Sciences, Engineering, and Medicine.

Liscovitch-Brauer, N., Alon, S., Porath, H.T., Elstein, B., Unger, R., Ziv, T., Admon, A., Levanon, E.Y., Rosenthal, J.J.C., and Eisenberg, E. (2017). Trade-off between Transcriptome Plasticity and

Genome Evolution in Cephalopods. *Cell* 169(2), 191-202.e111. doi: 10.1016/j.cell.2017.03.025.

Petrosino, G. (2015). *The transcriptional landscape of the nervous system of Octopus vulgaris.* PhD PhD Thesis, Università degli Studi di Napoli Federico II.

Sayers, E.W., Barrett, T., Benson, D.A., Bolton, E., Bryant, S.H., Canese, K., Chetvernin, V., Church, D.M., DiCuccio, M., Federhen, S., Feolo, M., Fingerman, I.M., Geer, L.Y., Helmberg, W., Kapustin, Y., Krasnov, S., Landsman, D., Lipman, D.J., Lu, Z., Madden, T.L., Madej, T., Maglott, D.R., Marchler-Bauer, A., Miller, V., Karsch-Mizrachi, I., Ostell, J., Panchenko, A., Phan, L., Pruitt, K.D., Schuler, G.D., Sequeira, E., Sherry, S.T., Shumway, M., Sirotkin, K., Slotta, D., Souvorov, A., Starchenko, G., Tatusova, T.A., Wagner, L., Wang, Y., Wilbur, W.J., Yaschenko, E., and Ye, J. (2012). Database resources of the National Center for Biotechnology Information. *Nucleic Acids Research* 40(D1), D13-D25. doi: 10.1093/nar/gkr1184.

Sievers, F., Wilm, A., Dineen, D., Gibson, T.J., Karplus, K., Li, W., Lopez, R., McWilliam, H., Remmert, M., and Söding, J. (2011). Fast, scalable generation of high - quality protein multiple sequence alignments using Clustal Omega. *Molecular Systems Biology* 7(1), 539.

UniProt Consortium, A. (2016). UniProt: the universal protein knowledgebase. *Nucleic Acids Research* 45(D1), D158-D169.

Zhang, X., Mao, Y., Huang, Z., Qu, M., Chen, J., Ding, S., Hong, J., and Sun, T. (2012). Transcriptome analysis of the *Octopus vulgaris* central nervous system. *PLoS ONE* 7(6), e40320.
